# TASOR expression in naive embryonic stem cells safeguards their developmental potential

**DOI:** 10.1016/j.celrep.2024.114887

**Published:** 2024-10-24

**Authors:** Carlos A. Pinzon-Arteaga, Ryan O’Hara, Alice Mazzagatti, Emily Ballard, Yingying Hu, Alex Pan, Daniel A. Schmitz, Yulei Wei, Masahiro Sakurai, Peter Ly, Laura A. Banaszynski, Jun Wu

**Affiliations:** 1 Department of Molecular Biology, University of Texas Southwestern Medical Center, Dallas, TX 75390, USA; 2 Hamon Center for Regenerative Science and Medicine, University of Texas Southwestern Medical Center, Dallas, TX 75390, USA; 3 Howard Hughes Medical Institute, Department of Cell Biology, Blavatnik Institute, Harvard Medical School, Boston, MA, USA; 4 Cecil H. and Ida Green Center for Reproductive Biology Sciences, Department of Obstetrics and Gynecology, Children’s Research Institute, University of Texas Southwestern Medical Center, Dallas, TX 75390, USA; 5 Department of Pathology, University of Texas Southwestern Medical Center, Dallas, TX 75390, USA; 6 St. Mark’s School of Texas, Dallas, TX 75230, USA; 7 State Key Laboratory of Animal Biotech Breeding, College of Biological Sciences, China Agricultural University, Beijing 100193, China; 8 Department of Cell Biology, Harold C. Simmons Comprehensive Cancer Center, University of Texas Southwestern Medical Center, Dallas, TX 75390, USA; 9 These authors contributed equally; 10 Lead contact

## Abstract

Pinzon and O’Hara et al. investigated the role of the transgene activation suppressor (TASOR), a component of the human silencing hub (HUSH) complex, in pluripotent stem cells. Their study uncovers a crucial role for TASOR in maintaining cell viability during the exit of naive pluripotency even though it is rapidly downregulated during this transition.

## SUMMARY

The seamless transition through stages of pluripotency relies on a balance between transcription factor networks and epigenetic mechanisms. Here, we reveal the crucial role of the transgene activation suppressor (TASOR), a component of the human silencing hub (HUSH) complex, in maintaining cell viability during the transition from naive to primed pluripotency. TASOR loss in naive pluripotent stem cells (PSCs) triggers replication stress, disrupts H3K9me3 heterochromatin, and impairs silencing of LINE-1 (L1) transposable elements, with more severe effects in primed PSCs. Notably, the survival of Tasor knockout PSCs during this transition can be restored by inhibiting caspase or deleting the mitochondrial antiviral signaling protein (MAVS). This suggests that unscheduled L1 expression activates an innate immune response, leading to cell death specifically in cells exiting naive pluripotency. Our findings highlight the importance of epigenetic programs established in naive pluripotency for normal development.

## Graphical abstract

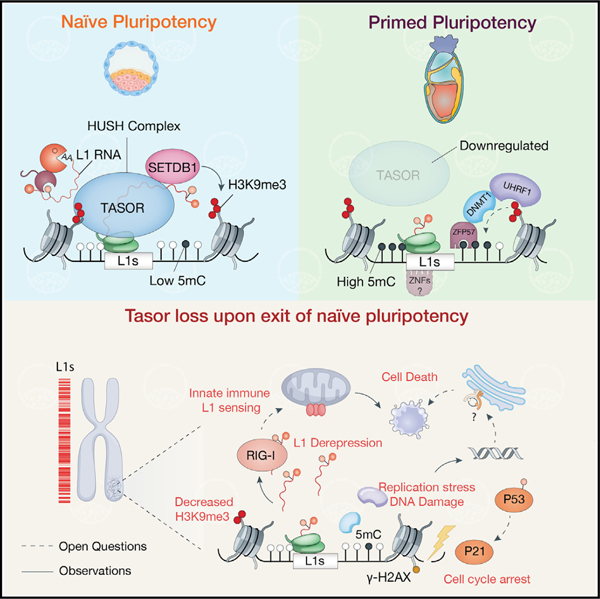

## INTRODUCTION

Mouse and human pluripotent stem cells (PSCs) cultured in the presence of the MEK 1/2 inhibitor PD0325901 exhibit low global DNA methylation, which is similar to the preimplantation epiblast and represent the naive state of pluripotency.^1–6^ Exiting the naive pluripotency state can be achieved by removing MEK inhibition or via direct exposure to fibroblast growth factor 2 (FGF2) and Activin A (FA), inducing mouse embryonic stem cells (ESCs) to differentiate into transient formative epiblast-like cells (EpiLCs),^7^ which can be further stabilized in culture as primed epiblast stem cells (EpiSCs).^8,9^ EpiSCs resemble the peri-gastrulation epiblast and are characterized by high levels of DNA methylation and an inactive X chromosome in female cells.^10^

Approximately half (~54%) of the human genome is composed of repeat sequences that include more than 630 long interspaced nuclear elements (LINEs or L1s).^11^ In somatic cells, L1s are typically silenced through DNA 5mC CpG methylation,^11^ yet in naive PSCs, L1s are transcribed^12^ as a consequence of the hypomethylated genome.^13^ The dysregulation of L1s has been linked to age-related disorders^14,15^ and carcinogeneis.^6,16^ Therefore, elucidating the mechanisms that safeguard the hypomethylated genome of naive PSCs against the activation of repetitive elements may enhance our understanding of the etiology underlying L1-associated disorders and help develop new therapeutic interventions.

The silencing of evolutionarily young L1 endogenous transposable elements in embryonic cells is mediated by the human silencing hub (HUSH) complex, which directs SETDB1 to deposit H3K9me3 at repeats and intronless mobile elements.^17–21^ TASOR is a component of the HUSH complex, along with M-phase phosphoprotein 8 (MPP8) and periphilin 1. Despite previous studies, TASOR’s function in early embryonic development is not fully understood. Here, we explore the role of TASOR in maintaining and exiting naive pluripotency through loss-of-function and epigenomic profiling studies.

## RESULTS

### TASOR loss results in massive cell death upon exit of naive pluripotency

**Figure 1.**
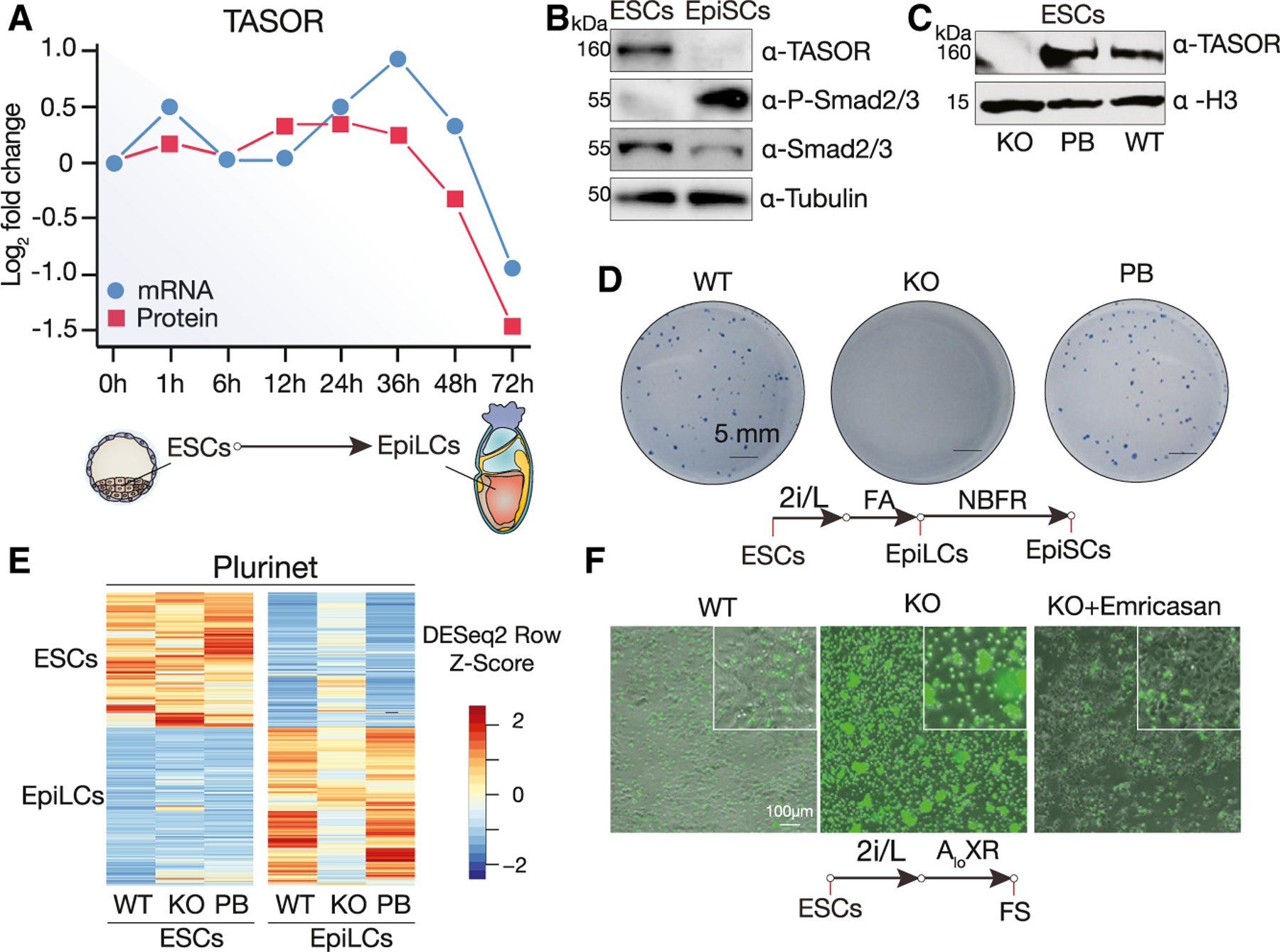
TASOR loss induces cell death during ESC differentiation (A) Diagram of Tasor mRNA and protein levels during mouse ESC-to-EpiLC transition from the Stem Cell Atlas dabase.^23^ (B) Western blot showing the expression of TASOR, P-SMAD2/3, SMAD2/3, and α-tubulin (loading control) of mouse ESCs cultured in 2i/L (naive), and EpiSCs cultured in NBFR (primed). (C) Western blot showing the expression of TASOR and histone H3 (H3; loading control) for wild type (WT), Tasor knockout (KO), and putback (PB) mouse ESCs. (D) Colony formation assay for naive ESC cells transitioned to primed EpiSCs. (E) Heatmap of differentially expressed pluripotency-related genes^26^ between mouse ESCs and EpiLCs in WT, *Tasor* KO, and PB cells. (F) SYTOX Green staining for cell death in mouse ESCs transitioning to the formative state (FS) and cultured for 72 h in AloXR.

Many epigenetic features associated with the pre- and post-implantation epiblast, including global DNA methylation patterns,^22^ can be recapitulated by cultured PSCs and PSC-derived embryo models (Figures S1A, S1B, S2A, and S2B). To identify epigenetic regulators of epiblast development, we performed bioinformatic analyses using published datasets^23^ and compared chromatin interacting proteins during the transition from mouse ESCs to EpiLCs. We found that the HUSH component *Tasor* is highly expressed in ESCs but rapidly downregulated at both the mRNA and protein levels upon differentiation to EpiLCs (Figure 1A). We confirmed that TASOR protein is indeed lost in mouse EpiSCs (Figure 1B) and human (Figure S2C) primed PSCs. This observation is particularly interesting given previous reports showing that a loss-of-function mutation in *Tasor* leads to an *in vivo* gastrulation defect resulting in embryonic lethality.^24,25^

To investigate the role of TASOR in naive pluripotency, we generated *Tasor* knockout (KO) mouse ESCs using CRISPR-Cas9^27^ (Figures 1C and S1C–S1E). *Tasor* KO ESCs maintained normal colony morphology and continued to express pluripotency markers when grown under the naive (2i/LIF) condition (Figures S1E and S1F). We next tested whether *Tasor* loss would affect the transition from naive ESCs (2i/LIF) to formative PSC conditions FAC (FGF2, Activin A and Chir99021, also known as “FTW,” for FGF, TGF and WNT pathway activation),^28^ and AloXR.^29^ Surprisingly, although both mRNA and protein are drastically downregulated upon exit of the naive state, we found that TASOR is required to establish formative PSCs, as only a few colonies are observed during the conversion in the absence of TASOR (Figures S1H–S1J). ESCs can transition to transient formative EpiLCs^7^ via exposure to FA (FGF2 and Activin A), and EpiLCs can be further differentiated and stabilized in culture as EpiSCs in the presence of FGF2 and a canonical WNT pathway inhibitor, IWR1(NBFR).^30^ EpiSCs closely resemble the ectoderm of the late-gastrula-stage embryo.^30,31^ Interestingly, we found that *Tasor* KO ESCs could transition to EpiLCs but largely failed to form colonies in the NBFR medium (Figure 1D). Notably, cellular differentiation phenotypes were rescued upon reintroduction of *Tasor* cDNA (*Tasor* putback [PB]) under both primed and formative conditions (Figures 1D and S1H–S1J).

We hypothesized that the observed differentiation defect in the absence of TASOR could be due to an inability to properly establish differentiation-specific transcriptional programs. However, RNA sequencing (RNA-seq) revealed that, despite some transcriptomic changes, *Tasor* loss did not prevent the differentiation of ESCs into EpiLCs (Figures 1E and S3A–S3H). This is evidenced by the upregulation of *Fgf5* and downregulation of *Nanog* (Figure S3C). Transcriptional changes were verified via RT-qPCR for the upregulation of the formative/primed pluripotency marker genes *Otx2* and *Fgf5* and the downregulation of *Prdm14* and *Tasor* (Figures S3E–S3H). To assess the differentiation potential of *Tasor* KO ESCs, we performed teratoma analysis. Despite being smaller than those from wild-type (WT) controls, teratomas derived from *Tasor* KO ESCs contained tissues from all three germ layers (Figures S1K–S1M). Overall, these results indicate that, despite compromised viability early in differentiation, mouse ESCs lacking TASOR are still capable of differentiating into cells from all three primary germ layers.

To determine whether the diminished colony-forming capacity of *Tasor* KO ESCs upon transitioning to formative pluripotency was due to cell death, we used SYTOX Green staining to quantify dead or dying cells following formative (AloXR) transition. In contrast to WT cells, we observed extensive cell death during the transition of *Tasor* KO ESCs (Figure 1F). Cell death can occur through several mechanisms, including apoptosis, pyroptosis, and necroptosis,^32^ or through a combination of these pathways, known as PANoptosis.^33^ A common feature of some of these pathways is the cleavage of cysteine-dependent aspartate-specific proteases (caspases). We thus applied the pan-caspase inhibitor emricasan and found that 5 μM emricasan significantly enhanced cell survival following formative transition (Figure 1F). Previous studies have demonstrated that *Tp53* deficiency can partially mitigate the gastrulation defect observed in *Tasor* mutant embryos.^25^ Thus, apoptosis is likely part of the cell death mechanism. These findings collectively indicate that TASOR is crucial for the transition from naive to formative and primed pluripotency states and that the loss of *Tasor* in mouse ESCs leads to a marked increase in cell death among formative and primed cells.

### TASOR loss leads to increased DNA damage and cell-cycle arrest in mouse ESCs

**Figure 2.**
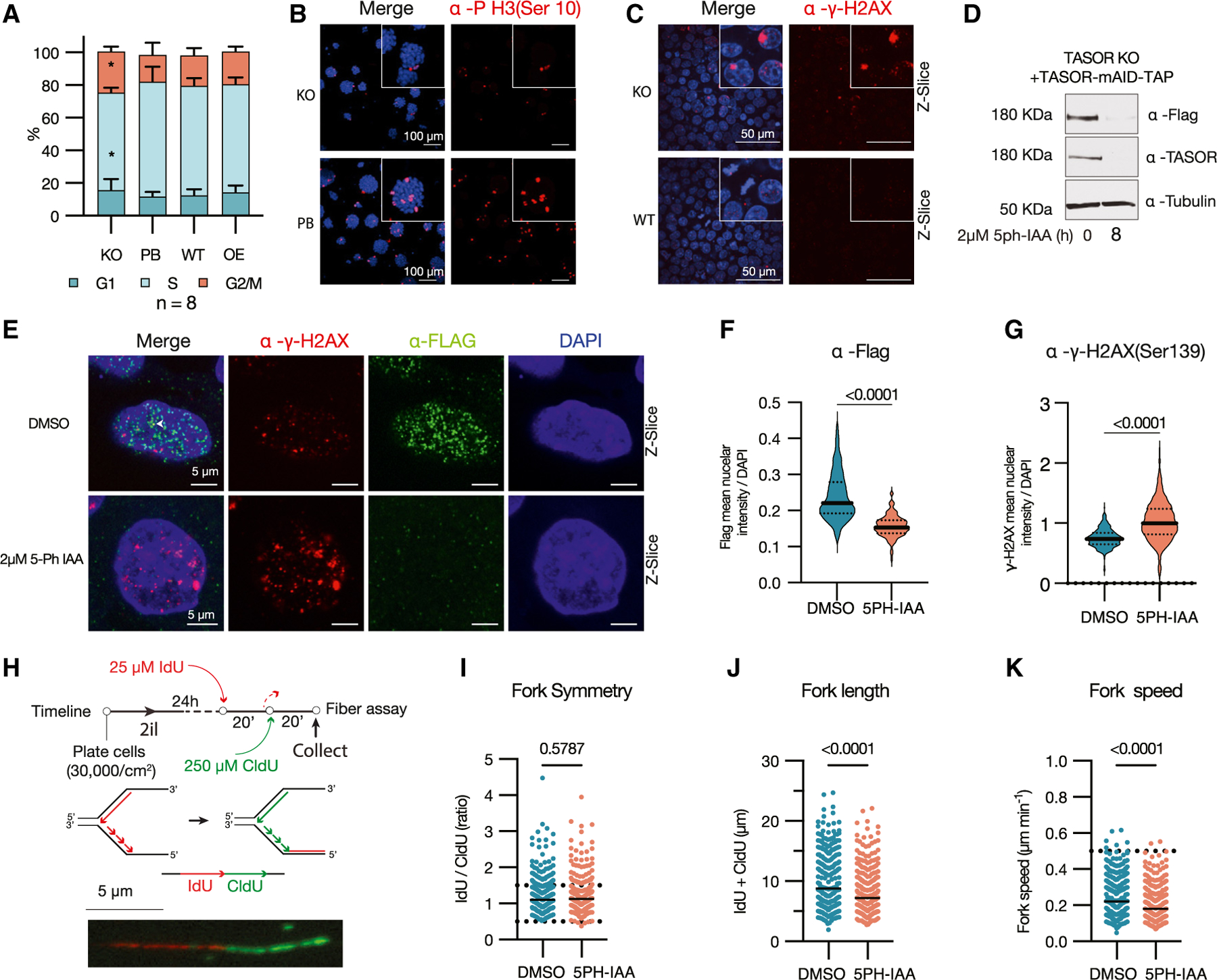
TASOR loss induces cell-cycle arrest, DNA damage, and DNA replication stress (A) Flow cytometry cell cycle analysis via EdU incorporation and DNA staining of *Tasor* KO, PB, WT, and overexpression (OE) mouse ESCs. Bars represent standard deviation (SD) among biological replicates. Kruskal-Wallis one-way ANOVA with Tukey’s HSD from 8 biological replicates, *adjusted *p* < 0.05. (B) Immunofluorescence staining for the mitosis marker phospho H3 (serine 10) for *Tasor* KO and PB mouse ESCs. Scale bar: 100 μm. (C) Immunofluorescence staining of WT and *Tasor* KO mouse ESCs for phosphoserine 139 of histone H2AX (γH2AX). Scale bar: 100 μm. (D) Western blot for TASOR 8 h after addition of 2 μM 5-ph-IAA. (E) Z-slice confocal immunofluorescence image for γH2AX in control DMSO or 5-Ph-IAA-treated cells. The white arrow indicates colocalization between FLAG and γH2AX. (F) Mean segmented nuclear intensity normalized to DAPI of FLAG Alexa Fluor 488. Unpaired t test from 2 biological replicates. (G) Mean segmented nuclear intensity normalized to DAPI of γH2AX Alexa Fluor 555. Unpaired t test from 2 biological replicates. (H) Diagram of DNA fiber assay with a representative image of a chromatin fiber. (I) Replication fork symmetry quantification of chromatin fibers in DMSO or 5-ph-IAA treated cells (*n* = 2). Kruskal-Wallis one-way ANOVA with Tukey’s HSD from 2 biological replicates. (J) Replication fork length quantification of chromatin fibers in DMSO or 5-ph-IAA-treated cells (*n* = 2). Kruskal-Wallis one-way ANOVA with Tukey’s HSD from 2 biological replicates. (K) Replication fork speed quantification of chromatin fibers in DMSO or 5-ph-IAA-treated cells (*n* = 2). Kruskal-Wallis one-way ANOVA with Tukey’s HSD from 2 biological replicates.

Previous studies have demonstrated that disrupting the HUSH complex leads to DNA damage and cell-cycle arrest.^34,35^ Consistently, the loss of TASOR in mouse ESCs resulted in a longer doubling time, which was reversed upon *Tasor* cDNA reintroduction (Figure S4A). Cell cycle analysis via EdU incorporation revealed that the increased doubling time is due to both an elongated G2/M phase and a shortened S phase (Figures 2A and S4B). Additionally, there was a decrease in the number of cells positive for the mitotic marker phospho (S10) H3 (H3S10ph) (Figures 2B, S4C, and S4D), indicating that the accumulation of cells in G2/M is likely caused by a G2 arrest. We investigated whether DNA damage might contribute to the observed G2 arrest. Our findings revealed that the absence of *Tasor* is associated with elevated levels of phospho (S139) gamma H2AX (γ-H2AX) (Figure 2C), along with increased phospho p53, and phospho (S2056) DNA-protein kinase catalytic subunit (DNA-PKcs) (Figure S4E). Additionally, RNA-seq indicated an increase in the expression of retinoblastoma (*Rb1*) in *Tasor* KO ESCs (Figure S4F). Notably, *Rb1* expression was further upregulated upon transition to EpiLCs along with the cyclin-dependent kinase inhibitor 1A (*Cdkn1a*, also known as *p21*) (Figure S4G), suggesting that activation of the p53-p21-Rb pathway^36^ might be driving the G2 cell-cycle arrest.

To elucidate the immediate impact of TASOR loss on DNA damage, we engineered a mouse ESC line containing *Oryza sativa* TIR1 (OsTIR1) F74G and a *Tasor* re-expression vector with a C-terminal mini auxin-inducible degron (mAID).^37,38^ Treatment with 2 μM 5-phenyl-indole-3-acetic acid (5-Ph-IAA) rapidly degraded TASOR (Figures 2D and S4H), resulting in a marked decrease in the nuclear signal of TASOR (Figures 2E and 2F), H3K9me3 (Figure S4J), phospho-H3 (Figure S4L), and MPP8 (Figure S4M) while leaving H3K27me3 levels unchanged (Figure S4N). These observations suggest that the acute loss of TASOR mirrors the effects of its chronic absence. Using this system, we confirmed a significant increase in γ-H2AX foci 24 h post TASOR depletion (Figures 2E and 2G). Further analysis through co-staining for FLAG-tagged TASOR, H3K9me3, and γ-H2AX, revealed that TASOR appeared as nuclear puncta and co-localized with some H3K9me3 and γ-H2AX foci (Figures 2E, S4I, and S4K). Given that H3K9me3 accumulates at stalled replication forks and DNA double-strand breaks^39,40^ and that its loss can compromise replication fork stability^41^ and double-stranded break repair,^40^ we sought to determine whether the increased γ-H2AX was due to replication fork instability. By labeling replication tracts with 5-chloro-2ʹ-deoxyuridine and 5-iodo-2ʹ-deoxyuridine and analyzing DNA fibers (Figure 2H), we found that TASOR depletion reduced both replication fork length and speed without affecting fork symmetry (Figures 2I–2K), pointing to replication fork stress in the absence of TASOR in mouse ESCs. Finally, by analyzing published genome-wide R loop profiling data in mouse ESCs,^42^ we found that R loops accumulate at L1 5ʹ promoter monomers, coinciding with TASOR and H3K9me3 occupancy (Figure S7G). The accumulation of R loops at L1 promoters following TASOR loss during DNA replication may be a key factor in DNA damage, likely due to collisions with the replication forks.^43^

### TASOR and DNA methylation silence L1 transcripts in mouse ESCs

As H3K9me3 is known to precede DNA methylation^44^ via a UHRF1-DNMT1 read and write mechanism,^44–46^ we wanted to evaluate the role of DNA methylation in cellular phenotypes observed upon TASOR loss. Previous studies have demonstrated that MEK-ERK inhibition leads to a dose-dependent decrease in DNA 5mC methylation in mouse ESCs, a process attributed to the loss of UHRF1 and reduced expression of the *de novo* methyltransferases *Dnmt3a*, *Dnmt3b*, and *Dnmt3l*.^47,48^ Accordingly, lowering the MEK1/2 inhibitor PD0325901 concentration from 1 μM to 0.2 μM in titrated 2i/L (t2i/L) increases global DNA methylation levels without altering the pluripotency state.^49^ Conversely, treatment with L-ascorbic acid (a form of vitamin C), a cofactor for Fe(II)/2-ketoglutarate-dependent dioxygenases, promotes DNA hypomethylation.^50,51^

We used the t2i/L culture condition or treatment with vitamin C to facilitate an increase (t2i/L) or decrease (vitamin C) in global DNA 5mC levels in mouse ESCs in the presence and absence of TASOR (Figures S1A, S1B, and S5A). We found that *Tasor KO* ESCs were more vulnerable to global DNA hypomethylation induced by vitamin C when compared to controls (WT, PB, and overexpression [OE] ESCs), resulting in a more pronounced increase in doubling time and cell-cycle arrest (Figures S5E–S5G). The addition of 100 μg/mL of vitamin C^4,52^ further exacerbated the de-repression of L1 ORF1 protein levels (Figures S5B–S5D) and led to an increase in segregation errors and chromosomal abnormalities (Figures S5H–S5O). Interestingly, when cultured in t2i/L, both WT and *Tasor* KO ESCs exhibited a subtle but noticeable increase in global H3K9me3 levels (Figures S5B and S5C) and a decrease in doubling time compared to those cultured under standard 2i/L conditions (Figure S5E). These findings demonstrate that culturing in t2i/L can partially mitigate the cell cycle phenotype of *Tasor* KO ESCs, likely by enforcing L1 silencing through increased DNA methylation. A similar compensatory response was observed in *Mpp8* KO ESCs grown under the serum/LIF condition.^53^

To determine the relationship of DNA methylation and TASOR-mediated H3K9me3 in L1 silencing in ESCs, we compared *Tasor* KO ESCs with triple *Dnmt* KO (*Dnmt*-3×KO) ESCs, which lack *Dnmt1*, *Dnmt3a*, and *Dnmt3b*. Immunostaining revealed that *Dnmt*-3xKO ESCs exhibited a loss of the DNA methylation marks 5mC and 5hmC (Figures S5P and S5Q) yet maintained observable levels of H3K9me3 and H3K27me3 (Figures S5R and S5S). Notably, *Dnmt*-3×KO ESCs did not phenocopy the decreased number of cells positive for H3S10ph (Figure S5T) nor the prolonged doubling time (Figures 2B and S5U) observed in *Tasor* KO ESCs. Additionally, upon culturing in AloXR for 48 h, we did not observe substantial cell death (Figure 4B), consistent with a previous report.^54^ However, *Dnmt*-3×KO ESCs exhibited a similar increase in L1 RNA transcripts as observed in *Tasor KO* ESCs (Figure 4C). These results suggest that, in mouse ESCs, both TASOR-mediated H3K9me3 and DNA methylation are critical for L1 repression. This contrasts with somatic cells, where DNA methylation plays a more dominant role, and L1 elements remain repressed through strong promoter methylation even in the absence of TASOR.^55,56^

### TASOR regulates steady-state LINE1 RNA transcripts

**Figure 3.**
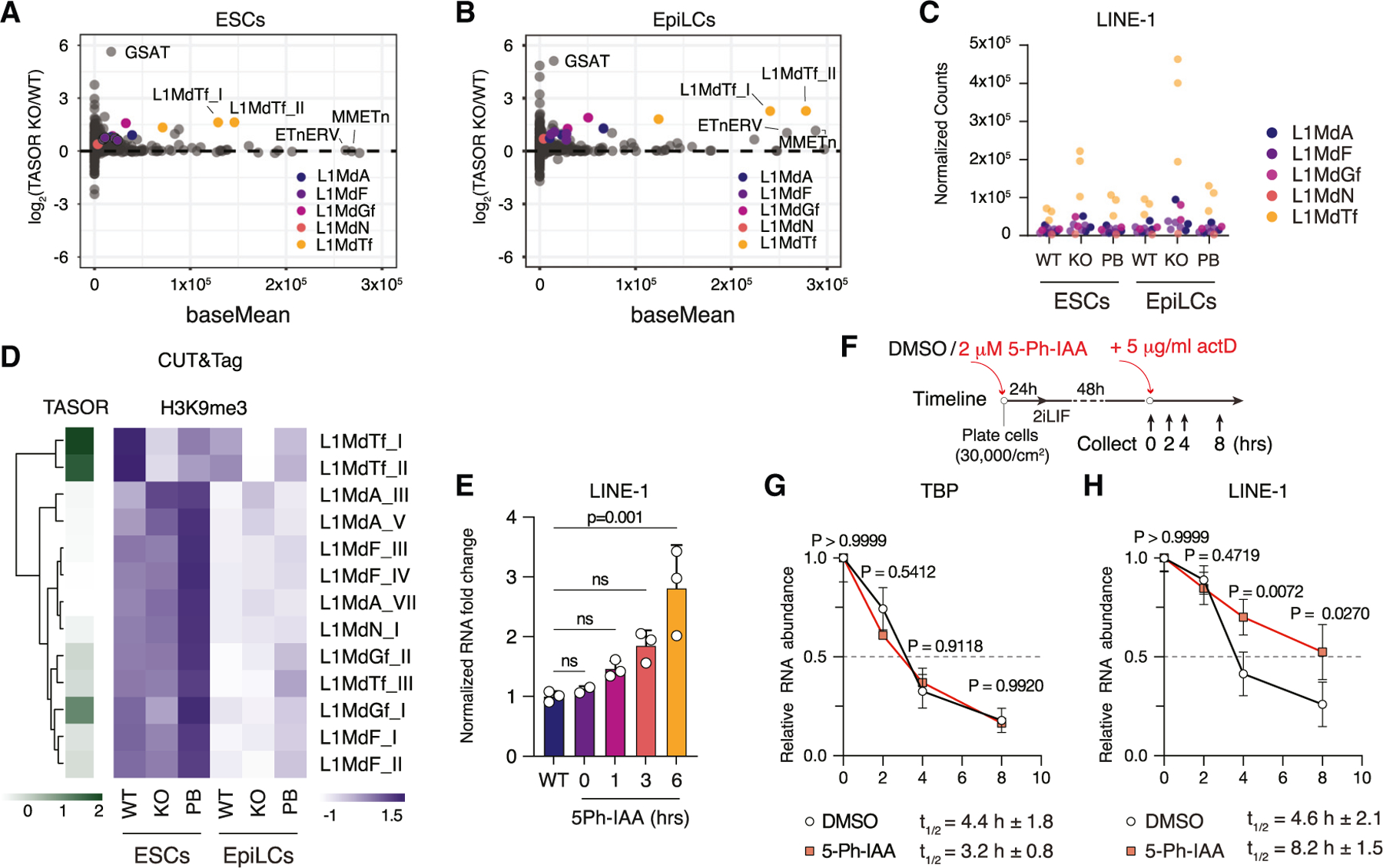
L1 RNA abundance and half-life is increased upon TASOR loss (A) MA plots for repeats showing log2 fold change of *Tasor* KO over WT mouse ESCs. (B) MA plots for repeats showing log2 fold change of *Tasor* KO over WT mouse EpiLCs. (C) Normalized average counts for LINE-1 (L1) subfamilies in mouse ESCs and EpiLCs for WT, *Tasor* KO, and PB. (D) Heatmap for CUT&Tag of TASOR-33Flag and H3K9me3 at L1 family members. (E) Time-course normalized RNA fold change via qPCR for L1 after Auxin treatment. Kruskal-Wallis one-way ANOVA with Tukey’s HSD from 2 or more biological replicates. Bars represent standard deviation (SD) among biological replicates. (F) Experimental diagram for measuring RNA half-life after actinomycin D (ActD) treatment. (G) Relative mRNA abundance after ActD treatment for TBP measured by RT-qPCR. Two-way ANOVA with Geisser-Greenhouse correction. Bars represent standard deviation (SD) among biological replicates. (H) Relative mRNA abundance after ActD treatment for L1 measured by RT-qPCR. Two-way ANOVA with Geisser-Greenhouse correction. Bars represent standard deviation (SD) among biological replicates.

The HUSH complex is well known for its role in L1 retrotransposon silencing.^57^ To investigate whether loss of retrotransposon silencing contributes to cell death in *Tasor* KO ESCs during the transition to the formative state, we first mined our RNA-seq data. Our analysis revealed that TASOR loss led to a significant increase in the steady-state levels of L1 RNA in ESCs, particularly within the evolutionarily young L1MdTf family of LINEs (Figure 3A). Upon transition to EpiLCs, the absence of TASOR resulted in an even more pronounced surge in L1 transcript abundance (Figure 3B), which was further confirmed by immunostaining (Figures S1E and S1P). During the formative transition, the L1MdTf subfamily was the most affected, with notable dysregulation also observed in the L1MdA and L1MdG subfamilies (Figures 3B and 3C). Beyond LINEs, modest yet significant increases in transcript abundance were observed for two endogenous retrovirus 2 (ERV2) subfamilies, MMETn (early transposon) and ETnERV, as well as for several satellite repeat subfamilies, including general satellites (GSAT_MM), centromeric satellite (CENSAT_MM), and minor satellite repeats (SYNREP_MM). These satellite repeat subfamilies (GSAT_MM, CENSAT_MM, SYNREP_MM) exhibited substantial fold changes to WT, albeit at relatively low transcript levels (Figures S6A and S6F–S6I). Given the most striking changes in transcript abundance observed in *Tasor* KO EpiLCs for LINEs, our further analyses focused on these elements.

The HUSH complex has been associated previously with the silencing of LINE elements through the deposition of H3K9me3.^17,18,58^ To investigate the impact of TASOR loss on H3K9me3 levels at LINE elements, we performed cleavage under targets and tagmentation (CUT&Tag)^59^ for FLAG (TASOR) and H3K9me3. Our analysis revealed that TASOR predominantly binds to the 5ʹ regulatory region of L1MdTf elements,^60^ with lesser binding observed across various other L1 subfamilies (Figures 3D and S6B). In comparison, H3K9me3 was highly enriched across a broad spectrum of repetitive elements, including LINEs, ERVs, and telomeric and satellite repeats (Figures S6F–S6I), with H3K9me3 peaks at L1MdTf elements frequently located near the 5ʹ end (Figure S6C). Following *Tasor* KO, a notable reduction in H3K9me3 was observed in both ESCs and EpiLCs, especially at L1MdTf subfamilies (Figures 3D and S6C). This decrease in H3K9me3 at L1MdTf elements was reversed upon reintroduction of *Tasor* cDNA (Figure S6C). Furthermore, immunostaining, flow cytometry, and immunoblot analyses also revealed a partial reduction in H3K9me3 levels in both mouse and human *Tasor* KO ESCs (Figures S1N, S2I, S2J, and S5R). *TASOR* KO human ESCs also showed increased upregulation of L1 transcripts (Figures S2L–S2O) and increased doubling time (Figures S2H) and had reduced differentiation capacity, as measured by blastoid formation efficiency (Figures S2P and S2Q). These results demonstrate TASOR’s critical role in establishing and/or maintaining H3K9me3 at the 5ʹ end of specific LINE subfamilies and highlight that the loss of TASOR and subsequent reduction of H3K9me3 are linked to increased LINE RNA abundance.

Since H3K9me3 is closely linked to gene silencing and pre-dominantly found in heterochromatin, we explored whether the loss of H3K9me3 at L1MdTf elements would result in increased chromatin accessibility. To this end, we performed an assay for transposase-accessible chromatin followed by sequencing (ATAC-seq).^61^ Contrary to the significant reduction of H3K9me3 at L1MdTf elements following *Tasor* KO, the increase in ATAC signal in *Tasor* KO ESCs and EpiLCs was only modest (Figure S6E). Interestingly, the transition of WT ESCs to EpiLCs resulted in both a higher ATAC signal and a decrease in H3K9me3 at L1MdTf without altering L1 RNA levels (Figures 3D and S6C–S6E). This observation suggests that, during this transition, alternative silencing mechanisms, such as DNA methylation, might come into play.

Motif analysis of ATAC peaks revealed that, upon transition to EpiLCs, ESCs showed reduced accessibility at motifs associated with pluripotency transcription factors (SOX2 and POU5F1), while EpiLCs showed increased accessibility at motifs associated with DNA methylation and imprinting (ZFP57) and higher-order chromatin organization (CCCTC-binfing factor, CTCF) (Figure S7A). In contrast, *Tasor* KO ESCs exhibited reduced accessibility at ZFP57 binding sites relative to WT cells (Figure S7B). Furthermore, during the transition, *Tasor* KO ESCs failed to decommission transcription factors from the KLF and POU families, including POU5F1 (also known as OCT4) (Figure S7C). These results suggest that, although *Tasor* KO ESCs could differentiate to EpiLCs, some transcriptional differences may arise from their inability to properly deactivate ESC-specific transcription networks (Figure 1E).

Interestingly, we noted the transcriptional dysregulation of various imprinted genes (Figure S7D), including notable changes in *Igf2* (Figures S7E and S7F), and observed that certain genes containing internal L1s were de-repressed in a manner dependent on the orientation and location of the L1 sequence (Figure S7G). Specifically, genes such as *Mrc1* and *Fsd1l* exhibited de-repression only in exons downstream of the L1 elements (Figures S3K and S7G–S7I). Additionally, *Tasor* KO ESCs showed upregulation in the expression of gene exons adjacent to L1 sequences (Figure S7J). This observation can potentially be explained by recent reports of L1s acting as “gene traps” during splicing events, leading to the creation of chimeric transcripts,^62,63^ or due to the disruption of H3K9me3 “spreading” mediated by the HUSH complex and MORC2 at these loci.^64,65^

Beyond its involvement in H3K9me3 deposition, the HUSH complex has also been implicated in the targeted degradation of L1 RNA via interactions with the nuclear exosome targeting (NEXT) and CCR4-NOT complexes.^21,66–68^ To study the effect of TASOR loss on L1 RNA stability, we utilized the auxin-inducible TASOR-mAID line. Treatment with auxin led to a rapid increase in L1 transcripts, coinciding with the depletion of TASOR protein (Figures 2D–2F and 3E). Using actinomycin D to halt further transcription, we tracked the persistence of selected RNAs over 8 h in the presence or absence of TASOR (Figure 3F). In control cells, the half-life of transcripts not typically targeted by be HUSH complex, such as TBP, remained unchanged (Figure 3G). Strikingly, auxin-induced TASOR depletion significantly increased the half-life of L1 RNA (t_1/2_ = 8.2 ± 1.5 h) compared to the DMSO-treated control (t_1/2_ = 4.6 ± 2.1 h) (Figure 3H), indicating that the observed increases in L1 RNA following TASOR loss are partly due to decreased degradation of L1 transcripts.

### An innate immune response mediates cell death during *Tasor* KO ESC-to-EpiLC transition

**Figure 4.**
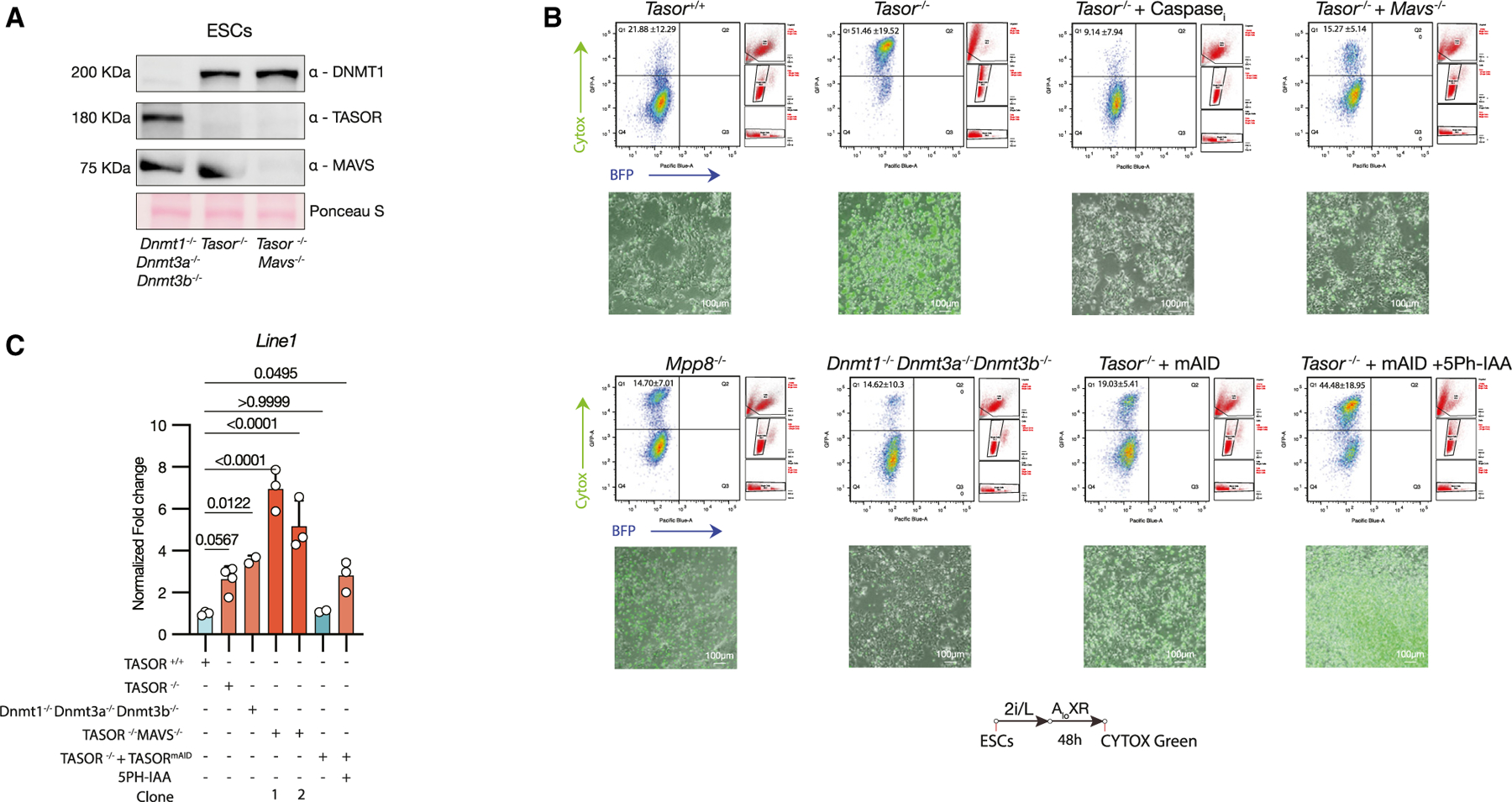
Cell death induced by TASOR loss is partially mediated by a MAVS innate immune response (A) Western blot analysis for DNMT1, TASOR, and MAVS in *Dnmt*33KO, *Tasor* KO, and *Tasor/Mavs* double KO (dKO) mouse ESCs. (B) Representative epifluorescence images for cell death using SYTOX Green staining (bottom) and flow cytometry analysis (top) after 48 h of AloXR-mediated formative stem cell conversion. Percentages are shown as mean +/− standard deviation (SD) (C) RT-qPCR analysis for L1 abundance in WT, *Tasor* KO, *Dnmt*33KO, *Tasor/Mavs* dKO mouse ESCs, and *Tasor* KO + TASOR^mAID^ mouse ESCs with and without 2 μM 5ph-IAA treatment. Kruskal-Wallis one-way ANOVA with Tukey’s HSD from 2 or more biological replicates. Bars represent standard deviation (SD) among biological replicates.

The HUSH complex plays a pivotal role in linking retrotransposon silencing with innate immunity, primarily through its control of L1 expression and the subsequent mitochondrial anti-viral signaling protein (MAVS)-dependent sensing of L1 RNA.^18,69^ In the context of cancer, disruptions to the HUSH complex leading to L1 dysregulation have been shown to trigger innate immune pathways, resulting in the death of cancer cells.^19,69^ We speculated whether the cell death observed during the transition from *Tasor* KO ESCs to EpiLCs could be due to an innate immune response. To test this, we first evaluated the expression of innate immune response genes upon acute depletion of TASOR via auxin and observed upregulation of *Irf7*, *Isg20*, *Cxcl10*, and *Ddx58* (Figure S8A). Native gel electrophoresis and western blotting revealed that IRF3 dimerization is more pronounced in *Tasor* KO ESCs compared to WT ESCs, indicating a more activated retinoic acid-inducible gene I-like receptor signaling pathway, where MAVS serves as the central hub.^70^ To further investigate, we generated *Mavs/Tasor* double KO (dKO) ESCs (Figure 4A). *Mavs/Tasor* dKO cells showed reduced IRF3 dimer formation compared to *Tasor* KO ESCs (Figure S8B). Strikingly, viability assessments using Cytox Green staining and flow cytometry analysis during AloXR-mediated formative transtion revealed that *Mavs/Tasor* dKO decreased the percentage of dead cells to levels similar to WT (Figures 4B and S8C). However, when *Mavs/Tasor* dKO ESCs transitioned through EpiLCs to EpiSCs, we observed diminished colony formation and alkaline phosphatase staining when compared to *Tasor* WT and PB, though the outcomes were still an improvement over *Tasor* KO ESCs (Figure S8D). Surprisingly, *Mavs/Tasor* dKO ESCs demonstrated a significantly reduced capacity to form teratomas compared to *Tasor* KO ESCs (Figures S8E and S8F), suggesting that, while *Mavs* KO mitigates cell death immediately following exit from naive pluripotency, its simultaneous loss with TASOR compromises cell differentiation and/or viability in later developmental stages. Unexpectedly, two separate *Mavs*/*Tasor* dKO clonal ESCs both showed markedly increased levels of L1 RNA when compared to *Tasor* KO (Figure 4C). Considering L1 RNA’s role in activating the MAVS-mediated innate immune pathway,^19,69^ this increase might stem from a subpopulation of cells that normally undergo apoptosis in *Tasor* KO but survive in the *Mavs/Tasor* dKO background. These results demonstrate that, while cell death during the transition from naive to formative/primed pluripotency is MAVS dependent, the protective effect of MAVS deficiency on *Tasor* KO mouse ESCs does not extend to later developmental stages.

Finally, to determine whether the observed phenotypes associated with TASOR loss were dependent on other components of the HUSH complex, we generated MPP8 KO ESCs (Figure S8G). Interestingly, the loss of MPP8 loss did not recapitulate the phenotypes observed in *Tasor* KO ESCs, such as reduced teratoma size (Figures S8E and S8F) or the increased cell death during the AloXR transition (Figure 4B). These findings underscore the critical role of TASOR in regulating the exit of naive pluripotency.

## DISCUSSION

Overall, our study demonstrates that *Tasor* is essential for cell viability during the transition from naive to formative and primed pluripotency even though TASOR itself is rapidly downregulated during this process. The loss of TASOR leads to increased DNA damage in the naive pluripotency state. Furthermore, TASOR loss results in reduced H3K9me3 heterochromatin formation at evolutionarily young L1s. L1 transcript levels are upregulated in *Tasor* KO mouse ESCs, potentially as a result of transcriptional upregulation due to loss of H3K9me3, but also because of increased RNA half-life in the absence of TASOR. Elevated L1 levels in *Tasor* KO mouse ESCs are likely recognized through the MAVS-dependent innate immune pathway, resulting in cell death during the transition from naive to formative/primed pluripotency.

Recent studies show that the HUSH complex interacts with the leading-strand DNA polymerase ε (POLE) complex, promoting the asymmetric transfer of H3K9me3 to the leading strand of the replication fork. This H3K9me3 asymmetry silences “head-on” orientation L1 expression in S phase of the cell cycle, and loss of TASOR or POLE results in DNA damage.^35^ Additionally, our analysis of recent genome-wide profiling of R loops in ESCs^42^ shows that R loops accumulate at L1 5ʹ promoters. We hypothesize that increased L1 transcript levels in the absence of TASOR may increase R loop accumulation at L1 promoters during DNA replication. Thus, TASOR-mediated heterochromatin might function to prevent replication-transcription conflicts, thereby protecting cells from DNA damage.^43,71,72^

The observed increase in chromosome segregation errors, micronuclei, and abnormal karyotype indicates that TASOR loss may lead to genomic instability through mechanisms beyond replication stress. The presence of TASOR at major satellite and centromeric repeats, together with the reduction of H3K9me3 following TASOR depletion and the concurrent gain of major satellite repeat RNA, as evidenced by RNA-seq and qPCR analyses, points to a potential effect on centromere integrity. This notion is supported by studies involving epigenetic remodeling through a TALE-demethylase targeted to centromeric repeats,^73^ which have shown a reduction in H3K9me3 levels, impaired HP1 recruitment, and subsequent impacts on chromosome segregation during mitosis.

One principal function of TASOR is in establishing H3K9me3-mediated silencing of its genomic targets. Interestingly, although H3K9me3 is significantly reduced at L1s in the absence of TASOR, transcriptional upregulation of L1s is more pronounced in Tasor KO EpiLCs compared to Tasor KO ESCs. This suggests that H3K9me3 at L1s is necessary for initiating silencing in ESCs, with the HUSH-mediated H3K9me3 laying the groundwork for additional silencing mechanisms, such as DNA methylation,^60,74^ which become more prominent in EpiLCs. Previous studies have demonstrated that H3K9me3 is crucial for initiating DNA CpG methylation and maintaining low levels of histone acetylation, both key features of heterochromatin.^44,75,76^ DNA methylation, in turn, has been shown to promote H3K9me3 at pericentromeric heterochromatin,^77^ indicating a coordinated interplay between these epigenetic marks. It is likely that multiple mechanisms coordinate this H3K9me3–5mC crosstalk.^45,46,75,78,79^ In a read- and-write model, UHRF1^46^ reads H3K9me3 to guide DNMT1, facilitating DNA methylation at these sites. Moreover, DNMT1 can directly recognize H3K9me3 using its tandem Tudor domain,^45,46^ and the HUSH1/2^80,81^ component MPP8 physically links the histone methyltransferases GLP/G9a with DNMT3A through its chromodomain.^75,79^ L1s and pericentromeric repeats are also silenced by the zinc-finger proteins ZNF512 and ZNF512B via the recruitment of SUV39H1/2 methyltransferases,^82^ providing a secondary mechanism that complements HUSH silencing. This might explain why H3K9me3 is significantly reduced but not entirely eliminated at L1s in Tasor KO ESCs.

To further explore the role of DNA methylation in L1 silencing in ESCs, both in the presence and absence of TASOR, we employed various cell culture conditions and genetic approaches to manipulate global 5mC levels. We found that hypomethylation induced by vitamin C treatment led to increased L1 expression in both WT and Tasor KO cells. Additionally, t2i/L treatment increased global 5mC levels and alleviated cell cycle defects in Tasor KO ESCs, making them indistinguishable from control cells under these conditions. Furthermore, Dnmt-3×KO ESCs exhibited L1 expression levels similar to those in Tasor KO ESCs, suggesting that both DNA methylation and H3K9me3 contribute to L1 silencing in ESCs. Our data collectively suggest that, in self-renewing ESCs, DNA methylation works in concert with TASOR-mediated H3K9me3 silencing. However, during differentiation, there appears to be a change in the silencing dynamics, where DNA methylation takes over as the main mechanism by which L1s are silenced.

Our findings demonstrate that, in addition to increases in L1 transcription upon loss of TASOR-mediated heterochromatin, TASOR loss increases L1 mRNA stability. HUSH is known to interact with the co-transcriptional termination machinery,^68^ the RNA deadenylase CCR4-CNOT complex (The carbon catabolite repression 4 (CCR4)–negative on TATA-less (NOT))scaffold protein CNOT1,^66^ and components of the NEXT complex.^83^ Increased L1 mRNA stability has been observed following the KO of the m6A demethylase FTO,^84^ and HUSH is known to interact with the m6A reader YTHDF2.^66,67^ Overall, HUSH appears to act as its yeast homolog, the RNA-induced transcriptional silencing complex,^85–87^ by recognizing the nascent RNA transcript,^88^ establishing H3K9me3 silencing, and ensuring that no productive transcript is produced by facilitating its degradation.

While loss of TASOR results in DNA damage and loss of heterochromatin at L1s and repeats in naive ESCs, we do not see evidence of substantial cell death until ESCs are transitioned to primed pluripotency (i.e., EpiSCs). Furthermore, TASOR loss triggers a P53-P21-Rb-mediated DNA damage response cell-cycle arrest, a phenomenon similarly observed in cancer cells,^43,72^ where L1 upregulation significantly slows cancer growth and progression.^34,43,89^ ESCs are immunologically different from somatic cells.^90^ Unlike differentiated cells, ESCs exhibit a much weaker response to cytoplasmic double-stranded RNA and produce minimal amounts of interferon β.^91^ This reduced innate immune response might serve as a protective mechanism, allowing ESCs to avoid immune-related cytotoxicity.^90^ Our results suggest that the inability to silence repetitive elements activates an “innate immune checkpoint,” leading to the elimination of cells that cannot suppress these elements during näıve-to-primed transition. This hypothesis is supported by findings that depletion of *Tasor*,^24^ *YTHDC1*,^92^ SETDB1,^93–95^ G9a/GLP,^96,97^ SUV39H1/2,^97–99^ *Mettl3*,^92^ or *Dicer*^100^ results in L1 de-repression and embryonic lethality and could explain why L1 derepression appears substantially higher in TASOR KO EpiLCs compared with ESCs. It is important to note that mice lacking FTO^101^ or MPP8^34^ are viable, likely due to compensatory mechanisms, as evidenced by increased TASOR binding in the absence of MPP8.^18,35^

In conclusion, our findings underscore TASOR’s critical role in establishing chromatin states that allow the exit from naive pluripotency and reveal the intricate relationship between epigenetic regulation and innate immunity during embryonic development.

### Limitations of the study

While this study consolidates many previously published observations of TASOR function and extends them to the context of PSCs, it has several limitations. First, although TASOR loss leads to increased DNA damage and unscheduled L1 expression, the underlying mechanisms remain unclear. Specifically, it is not well understood why TASOR deficiency induces DNA damage or how TASOR-mediated heterochromatin prevents replication-transcription conflicts at L1 promoters. Additionally, while elevated L1 levels in TASOR KO cells may activate the MAVS-dependent innate immune pathway, the precise relationship between L1 derepression and the activation of innate immunity requires further investigation. Another limitation is the lack of understanding of why TASOR loss triggers cell death only during the transition to primed pluripotency. The hypothesis that innate immune pathways become active during this transition and contribute to cell death remains speculative and untested. The study also emphasizes the roles of H3K9me3 and DNA methylation in L1 silencing but leaves unanswered questions about the interplay between these epigenetic marks and the shift in silencing dynamics during differentiation. Furthermore, although TASOR loss results in increased L1 mRNA stability, the role of TASOR in RNA decay pathways—such as its interactions with the CCR4-CNOT complex and the NEXT complex—needs further clarification. Finally, while the study suggests a link between TASOR loss and genomic instability beyond replication stress, such as effects on centromere integrity, the specific contributions of these pathways to the observed chromosomal abnormalities are not fully explored. Further research is needed to elucidate TASOR’s multifaceted roles in maintaining genome stability and cell survival during the exit from naive pluripotency.

## RESOURCE AVAILABILITY

### Lead contact

Request for further information and resources should be directed to and will be fulfilled by the lead contact, Jun Wu (jun2.wu@utsouthwestern.edu).

### Materials availability

Plasmids and cell lines generated in this study are available upon request to the lead contact with a completed materials transfer agreement.

### Data and code availability

All datasets were deposited in the NCBI Gene Expression Omnibus using the following accession numbers: ATAC-seq, GEO: GSE262193; CUT&TAG, GEO: GSE262194; and RNA-seq, GEO: GSE262195. The CpG tracts where obtained from Yang et al.^102^ The R loop tracks were obtained from Wulfridge et al.^42^ The ZNF-512 tracks where obtained from Ma et al.^82^

## STAR★METHODS

### EXPERIMENTAL MODEL AND STUDY PARTICIPANT DETAILS

#### Culture of mouse embryonic stem cells (ESC)

Male and female mouse embryonic stem cells where derived in house from C57BL/6 genetic background. All cell cultures were performed in N2B27 basal media. This media was prepared using the following (250mL): 125 mL DMEM/F12 (Invitrogen), 125 mL neurobasal medium (Invitrogen), 1x N2 supplement (5mL, Invitrogen), 1x B27 supplement (10mL, Invitrogen), 1× GlutaMAX (2.5mL, Gibco), 1× nonessential amino acids (2.5mL, Gibco), 0.1 mM β-mercaptoethanol (Gibco), 1% fatty acid free BSA, and 2.5 μg/ml of prophylactic plasmocin (InvivoGen) to prevent mycoplasma contamination during maintenance of the cells but was removed before any experimental assay (InvivoGen).

All cell culture was performed as following: cells were washed with 1xPBS and dissociated with TrypLE (Thermo Fisher) for 3 min at 37°C; cells were then collected with 0.05% BSA in DMEM-F12 (Thermo Fisher) and centrifuged at 1000xg for 3 min and resuspended in 1mL of media per 9.6cm^2^. Each passage cells were counted using Countess II (Thermo Fisher) and plated at a density of 15,000 cells/cm^2^, at this plating ratio cells were passaged every 4 days. Fresh media (2mL per 9.6 cm^2^) was added during the first two days, and on days 3 and 4 the media amount was doubled. Cell cultures were maintained in a 37°C humidified in incubator with 5% CO_2_. Cells were cryopreserved in CryoStor CS10 (Biolife solutions) at 0.5×10^6 cells per mL in −80°C, using in CoolCell freezing containers (Corning). The following day, cells were moved for long-term storage in liquid nitrogen.

Naive mouse ESCs (naive ESCs) were cultured feeder free, on Geltrex (Gibco) coated cell culture dishes in 2i/L media. 2i/L media was prepared in N2B27 basal media with the addition of the following small molecules and cytokines: 1μM PD0325901 (MEK 1/2 inhibitor, Selleckchem), 3μM CHIR 99021 (WNT agonist via GSK3 α/β inhibition, Selleckchem) and in the presence of the STAT3 agonist, leukemia inhibitory factor (LIF, 10 ng/ml).

#### Culture of epiblast like stem cells (EpiLCs), epiblast stem cells (EpiSCs), and colony clonogenicity formation assays

Mouse epiblast like stem cells (EpiLCs), were transitioned from 2i/L cultures by plating the cells on Geltrex (Gibco) coated cell culture dishes at a density of 15,000 cells/cm^2^ in N2B27 based FA media containing 12 ng/ml of FGF2 and 20 ng/ml of Activin A for 48 h. Media was replaced every 24 h. Mouse epiblast stem cells (EpiSCs) were transitioned from EpiLCs by plating the cells on mitotically inactivated mouse embryonic fibroblast (MEF) on 0.1% gelatin coated cell culture dishes at a density of 15,000 cells/cm^2^ in N2B27-based NBFR media containing 20 ng/ml of FGF2 and 2.5mM of the WNT antagonist IWR1 (Selleckchem). Media was replaced every 24 h. For colony clonogenicity assays, cells were plated at 500–5000 cells/cm^2^. Once colonies had grown (6–15 days), cells were stained with an alkaline phosphatase staining kit following manufactures instructions (Abcam), or with Coomassie brilliant blue (Thermo).

#### Culturing of human naive PSCs

Naive WIBR3 human ES cells were obtained from R. Jaenisch and T. Theunissen.^103^ Primed human PSC were cultured in MEFs on 0.1% gelatin-coated cell culture dishes on N2B27 based media as described above. Primed cells were cultured on irradiated mouse embryonic fibroblasts in NBFR (Table S1) supplemented with 20 ng/ml of LIF and 20 ng/ml of Activin A. Primed cell lines were reset to the naive state following a previously described protocol^4104^. Briefly, 20,000 cells/cm^2^ primed cells were treated with 1μM PD0325901(Selleckchem), 1mM Valproic acid (VPA, Medchemexpress), 20 ng/ml of leukemia inhibitory factor (LIF, Peprotech) and CEPT cocktail [50nM Chroman 1 (MedChem Express), 5μM Emricasan (Selleckchem), 1X polyamine supplement (Sigma), and 0.7 μM TransISRIB (Tocris)], for 3 days. Then, media was changed to a modified 5i/L/A medium.

All naive human ESCs were cultured on Geltrex-coated dishes. Briefly, 25,000 cells/cm^2^ naive PSCs were plated into Geltrex-coated cell culture plates in the modified 5i/L/A medium.^104^ The cells were passaged as described above with 1xCEPT cocktail^105^ [50nM Chroman 1 (MedChem Express), 5 μM Emricasan (Selleckchem), 1X polyamine supplement (Sigma), and 0.7 μM TransISRIB (Tocris)], for 12 h. The modified 5i/L/A medium was prepared using N2B27 basal media with the addition of 0.5% KSR, 50 μg/mL of bovine serum albumin (BSA, Sigma) and the following small molecules and cytokines: 1μM PD0325901 (Selleckchem), 0.5μM IM-12 (Enzo), 0.5μM SB590885 (R&D systems), 1μM WH-4–023 (Selleckchem), 20 ng mL—1 recombinant human LIF (Peprotech), 10 ng mL—1 Activin A (Peprotech) and 5μM Y-27632 (Selleckchem)., 2μM XAV939 (MedChem Express) and 2 μM Gö6983 (MedChem Express). Naive ESCs were cultured for a minimum of 10 days in the modified 5i/L/A before any experiment. Naive human ESCs were never exposed to serum.

### METHOD DETAILS

#### Generation of blastoids from human naive PSCs

Blastoid generation was performed as described by Yu; et al.^104,106^ 5iLA naive human PSCs were dissociated into single cells by incubation with TrypLE (Thermo Fisher) for 3–5 min at 37°C. Cells were collected with 0.1% BSA in DMEM-F12 medium and centrifugated at 1000 rpm (approx. 200xg) for 3 min in a swing bucket centrifuge (Legend RT+, Thermo Fisher) and recovered in 5iLA medium with 1xCEPT and 10U/ml of DNase I (Thermo Fisher) and incubated at room temperature for 15min. Cells were then passed through a 20-μm cell strainer (Pluriselect). To select for viable cells and exclude dead cells and cell debris, cells were carefully layered on top of 10mL of 0.1% BSA DMEM-F12 in a 45° angle in a 15mL conical centrifuge tube and centrifuged at 300 rpm for 10 min. Supernatant was removed and pelleted cells were re-suspended and manually counted in a Neubauer counting chamber. Meanwhile, AggreWell-400 (STEMCELL Technologies) plate was prepared according to the manufacturer’s instructions. In brief, 500μL of anti-adherence solution (STEMCELL Technologies) was added to each well, the plate was centrifuged at 1500 x g for 5 min, and then incubated at room temperature for a minimum of 45 min. The corresponding number of cells (32 cells/microwell) were washed and resuspended in 1mL eHDM (3μM Chir99021, 10 ng/ml FGF2–3G, 20 ng/ml Activin A) with 1xCETP and seeded into one well of a precoated AggreWell-400 24-well plate in 1mL of media. Each well was carefully mixed using a P200 pipette and the plate was left alone for 15 min to ensure equal distribution of the cells inside the well. The plate was centrifuged at 1000 x g for 3 min and cultured at 37°C in 5% CO_2_ and 5% O_2_. The day of cell plating was designated as day 0. eHDM was completely changed to eTDM on day 1 by carefully removing as much eHDM as possible without disturbing the aggregates by tilting the plate in a 45° angle, each well was washed once with 200μL of eTDM, finally 1 mL of eTDM was slowly added 200 μL at a time. On the remaining days, fresh eTDM with fresh LPA was half changed every day. The human blastoids usually formed after four days of culture in eTDM. All blastoids were manually isolated using a mouth pipette under a stereomicroscope for downstream experiments. For eHDM and eTDM recipe see Table S1.

#### Immunofluorescent staining

Samples (cells, and blastoids) were fixed with 4% paraformaldehyde (PFA) in 1xDPBS with 0.1% PVA for 20 min at room temperature, washed in wash buffer (0.1% Triton X-100, 5% BSA in 1xDPBS) for 15 min and permeabilized with 0.1–1% Triton X-100 in PBS for 1 h. For 5mC/5hmC staining samples were treated with 4N HCL for 15 min, and then neutralized with 100mM Tris-HCL pH 8.0 for 30 min. Samples were washed 3 times in wash buffer for 5 min and then blocked with blocking buffer (PBS containing 5% Donkey serum, 5% BSA, and 0.1% Triton X-100) at room temperature for 1 h, or overnight at 4°C.

Primary antibodies were diluted in blocking buffer. Blastoids were incubated in primary antibodies in U bottom 96 well plate for 2 h at room temperature or overnight at 4°C. Samples were washed three times for 15 min with wash buffer, and incubated with fluorescent –dye-conjugated secondary antibodies (AF-488, AF-555 or AF-647, Invitrogen) diluted in blocking buffer (1:300 dilution) for 2 h at room temperature or overnight at 4°C. Samples were washed three times with wash buffer. Finally, cells were counterstained with 300nM 4ʹ,6-diamidino-2-phenylindole (DAPI) solution at room temperature for 20 min.

#### Imaging

Phase contrast images were taken using a hybrid microscope (Echo Laboratories, CA) equipped with objective ×2/0.06 numerical aperture (NA) air, x4/0.13 NA air, x10/0.7 NA air and 20x/0.05 NA air. Fluorescence imaging was performed on 8 or 96 well μ-bid (Ibidi) on a Nikon CSU-W1 spinning-disk super-resolution by optical pixel reassignment (SoRa) confocal microscope with objectives ×4/0.13 NA, a working distance (WD) of 17.1nm, air; ×20/0.45 NA, WD 8.9–6.9 nm, air; ×40/0.6 NA, WD 3.6–2.85 nm, air.

#### Imaging analysis

All Imaging experiments were repeated at least twice, with consistent results. In the figure captions, n denotes the number of biological repeats. Raw images were first processed in Fiji^107^ to create maximal intensity projection (MIP) and an export of representative images. Nuclear localized fluorescence intensity was quantification was made using Imaris (v10, Oxford) XT module and spots colocalization tool. Nuclear localized fluorescence intensity was computed for each cell in each field, and the value was then normalized to the DAPI intensity of the same cell. Intensity values of all cells were plotted as channel intensity over DAPI intensity for the same cell with mean ± s.d. GATA6 positive cells were selected and separated from negative cells using the spot colocalization tool. Epiblast cells were calculated as GATA6 negative spots. Trophectoderm and hypoblast cells as GATA6 positive spots.

#### Western blotting

A minimum of 1×10^^^6 cells were harvested by centrifugation and lysed in RIPA lysis buffer (150mM NaCL, 1% Nonidet P-40, 0.5% Sodium deoxycholate (DOC), 0.1% SDS, 50mM Tris-HCL) supplemented with 1mM PMSF, 2mM MgCl_2_, 1x Halt complete protease inhibitor cocktail (Thermo Fisher Scientific) and 1x Halt phosphatase inhibitor cocktail (Thermo Fisher Scientific). Cell lysates were incubated with 10μL of benzonase (Sigma) per 100μL of RIPA buffer, for 15minutes at room temperature. Lysates were quantified using PIERCE BCA protein assay kit (Thermo Fisher Scientific) as per manufacturer instructions and absorbance was measured at 562nm using a SpectraMax iD3 plate reader (Molecular Devices). Protein concentrations were normalized to the lowest sample. Samples were denatured with Laemmli buffer (0.05M Tris-HCl at pH 6.8, 1% SDS, 10% glycerol, 0.1% β-mercaptoethanol) by boiling for 10 min. 30μg of total protein were resolved using SDS-PAGE followed by transfer to PVDF membranes. TASOR and MPP8 where resolved in 5% SDS-PAGE 1.5mm gels with a minimun loading of 30μg of cell lysate. Wet transfer was performed at a constant current of 375 mA for 1 h, which corresponds to 0.0324 mA/mm^3^ of gel for a 1mm standar gel. Transfer was visualized using Ponceau S staining solution (0.5% w/v Ponceau S, 1% acetic acid). Membranes were cut and washed with TBS with 0.1% Tween (TBS-T) and blocking for 1 h with 5% MILK (BSA for phosphorylation specific antibodies) in TBS-T. Membranes were then incubated with the corresponding primary antibodies (key resources table). Immunoreactive bands were visualized using HRP conjugated secondary antibodies (key resources table), and incubated with chemiluminescence substrate (Pierce ECL western substrate, Thermo Fisher Scientific) and exposed to X-ray film or a ChemiDoc imaging system (BioRad).

#### Teratoma tumor formation assay

Mouse ESCs were suspended in a 1:1 mixture of Matrigel and DMEM/F12 medium at a concentration of 1×10^7^ cells/mL. 100 μL of each cell mixture (1×10^6^ cells per tumor) was injected subcutaneously into the flanks of immunodeficient NOD/SCID mice. After 4 weeks, tumors were dissected, weighed, and fixed in 4% paraformaldehyde for 48 h. Fixed tumor samples were submitted to the UT Southwestern Histopathology Core Facility for paraffin embedding and sectioning. Embedded and sectioned teratomas were stained with hematoxylin and eosin for tissue identification.

#### Cell cycle analysis and flow cytometry

Approximately 1×10^^^6 cells were treated with 10 μM EdU for 20 min at 37°C. Cells were harvested and fixed in ice-cold 96% methanol cells were permeabilized for 15 min using saponin and stained using the Click-iT Edu Alexa Fluor 488 flow cytometry assay kit (Thermo) as per manufacturing instructions, with DNA was counter staining with FxCycle Far Red with addition of RNAse (Thermo). Flow cytometry was performed using the appropriate unstained and single stain controls in a DBiosciences LSR II flow cytometer and analyzed using Flow Jo. Gating Strategy to determine cell cycle stages is shown in Figure S4B.

#### IRF3 dimerization assay

Cell pellets were resuspended in a modified RIPA buffer (50mM Tris Ph 8.0, 150mM NaCl, 1% NP40, 5% glycerol, 10mM sodium Fluoride, 0.4mM ETA, 1mM PMSF, 1xProtease inhibitor cocktail, 1xPhosphatase inhibitor cocktail), incubated on ICE for 30 min and centrifuge at 13,000xg for 10 min. Supernatant protein was quantified using the Pierce BCA quantification kit using a 1:2 dilution and absorbance at 563nm was detected in a spectrophotometer. Protein was flash frozen in liquid nitrogen and stored at −80°C. A 1.5mm non-denaturing 6% acrylamide/bis-acrylamide (29:1) gel without SDS at 4°C with the inner chamber buffer containing 25mM Tris-HCL pH 8.4, 192mM glycine and 1% deoxycholic acid in dH2O and the outer chamber containing 25mM Tris-HCl pH 8.4 and 192mM Glycine in dH2O was pre ran for 30 min on ice at constant 40 mA. 50μg of total protein was mixed with 2x loading dye (125mM Tris-HCL 6.8, 30% glycerol 0.1% Bromophenol blue in dH20) and ran at 40mA until the migration of the bromophenol blue exited the gel. The gel was washed for 15 min in 1x SDS-PAGE running buffer and transferred on ice to a PDVF membrane in 1x transfer buffer with 5% methanol at 375mA for 2 h.

#### 5-mC DOT blot assay

Dot blot analysis was made as described in Blaschke et al.^4^ with modifications, samples genomic DNA was purified from 1×10^6^ cells using the DNeasy blood & tissue Kit (Quiagen). DNA was eluted in 10mM Tris HCl pH 8 and quantified using a spectrophotometer. 2μg of DNA samples were denatured in 0.4M NaOH, 10mM EDTA at 100°C for 10 min, and neutralized by adding an equal volume of icecold 2M ammonium acetate pH 7.0 and, and then serially diluted 2-fold. Nitrocellulose membranes were pre wetted in 6xSSC, diluted DNA samples were spotted on a nitrocellulose membrane using a Bio-Dot microfiltration apparatus (Bio-Rad). The blotted membrane was washed in 2× SSC buffer, dried at 80°C for 5 min, and UV cross-linked at 120,000 μJ/cm^2^. The membrane was then blocked in 5% milk in TBS-T for 1h at room temperature. Mouse anti-5-methylcytosine monoclonal antibody (Active Motif, 1:500) was added for 2 h at room temperature. The membrane was washed for 10 min three times in TBS-T, and then incubated with HRP-conjugated goat anti-mouse immunoglobulin-G (IgG) (Thermo, 1:10,000) for 1h at room temperature. The membrane was then washed for 10 min three times in TBS-T and visualized with chemiluminescence substrate (Pierce ECL western substrate, Thermo Fisher Scientific) and a ChemiDoc imaging system (BioRad).

#### Cloning of TASOR by overlapping PCR

TASOR was amplified by overlapping PCR, three primer sets were designed with overlapping sections,100ng of Genomic DNA was used per PCR reaction with primeSTAR GXL DNA polymerase (Takara Bio) using a touchdown PCR for the first 10 cycles from 72 to 60 followed by 35–40 cycles at the proper annealing temperature (Tm −2°C) and extension 68°C 30 s/kb or 72°C 15 s/kb and purified using a PCR purification KIT (Qiagen). Equimolar amounts of PCR products were mixed and a PCR was made with a primeSTAR GXL DNA polymerase (Takara Bio) without primers for the first 10 cycles using the following thermocycler conditions (95°C 3min, 98°C 10s, 60°C 30s, 68°C 5min, go to 2 15x, followed by the addition of the forward and reverse primers (0.5μM ea) and the reaction continued as a normal PCR for the next 20 cycles. Reaction products were gel purified and cloned into the expression vector via Gibson assembly. Vectors were sequenced using nanopore sequencing sanger sequencing at repetitive sites (Eurofins genomics). Primers are listed in Table S2.

#### Auxin induced degradation of TASOR

Auxin-inducible degradation of TASOR was made using the auxin inducible degron 2 technology.^37^ For this mouse TASOR was cloned via Gibson assembly into a custom vector, expressing puromycin selection under a cytomegalovirus (CMV) enhancer fused to the chicken beta-actin promoter (CAG) promoter, a T2A sequence and TASOR with a C-terminal mini auxin-inducible degron (mAID) and a strep-strep-3xFlag tandem affinity purification (TAP). Oryza sativa TIR1 (OsTIR1) F74G was cloned downstream of the blasticidin resistance gene driven by a CAG promoter using a T2A sequence. Vectors were sequenced using nanopore sequencing Sanger sequencing at repetitive sites (Eurofins genomics). Cell lines were generated in a sequential manner via random integration and antibiotic selection with 5 μg/ml of blasticidin and 1 μg/ml of puromycin. Expression of TASOR and OsTIR1 was confirmed via qPCR and Flag immunofluorescence. Degradation was induced via the addition of 2μM 5ph-IAA, fresh media with 5ph-IAA was replaced every 12 h. For mRNA half-live experiments, transcriptional inhibition was made with 5 μg/ml of Actinomycin D.

#### Visualization of RNA-seq and CUT&Tag data

The transcriptome and ChIP-seq datasets were visualized using Integrative Genomics Viewer (IGV, version 2.3.88.^108^ Heatmaps and volcano plots were generated using R Statistical Software and the following R packages ggplot2, DESeq2m heatmap3, RcolorBrewer.

#### CRISPR-Cas9 mediated gene knockout

CRISPR-Cas9 sgRNAs^27^ were design as previously descrived,^109^ briefly all DNA sequences were manipulated using Benchling, sgRNAs were designed using Benchling with guide cleavage efficiency made with the WU-CRISPR tool.^110^ Guides with minimal off targets and high cleavage efficiency were chosen. Each guide and its complementary sequence were ordered as synthetic 25 nm oligos from (Thermo Fisher) with attached BbsI cloning sites: Sense: 5ʹ–CACCGNNNNNNNNNNNNNNNNNNN–3ʹ and antisense: 3ʹ–CNNNNNNNNNNNNNNNNNNNCAAA–5. Guides were cloned via golden gate assembly as described in Konnermann, S. et al.,^111^ with modifications. Briefly, 100pmol of each complementary oligo were phosphorylated using T4 PNK with T4 ligase buffer (contains ATP) for 30 min at 37°C, then oligos were annealed by denaturing at 95°C for 5 min and then slowly cooled down using a ramp of 5°C per minute up to 25°C. Phosphorylated and annealed oligos were diluted 1:10 and a Golden Gate reaction was setup with 1x rapid ligase (Roche), 10 units of BbsI enzyme, T7 DNA ligase (Roche) 25ng of backbone vector, reaction was run for 15 cycles of digestion at 37°C for 5 min and ligation at 20°C for 5 min. 2 μL of the golden gate reaction were transformed into competent cells. After confirmation via sanger sequencing, maxiprep of sgRNAs were made with the Purelink Hipure plasmid maxiprep kit (Thermo) and DNA was eluted in 10mM Tris-HCl at a concentration of 2μg/mL. Cells were transfected using a NEPA21 electroporator (Nepa Gene) using 4 μg of total DNA per 1×10^^^6 cells. Conditions. Four “poring pulses” were applied (150 V, 3.0 ms, interval 50 ms, 10% voltage decay, polarity+), followed by 5 “transfer pulses” (5 V, 50 ms, interval 50 ms, 40% voltage decay; alternating + and − polarity). Cells were rapidly placed in pre warmed culture media to recover. Cells were FACS sorted 24 to 72h after transfection, the top 20% of selection marker positive cells was sorted and collected. Cells were plated at a density of 1000 cells per 9.6cm^2^. Single cell colonies were marked with an object maker (Nikon) and manually picked under a microscope in a laminar flow hood. 50 percent of recovered cells was lysed in 50μL of Quickextract DNA extraction solution (Biosearch) and genotyped via PCR. Positive clones were expanded and PCR again to ensure no integration of the CAS9 backbone. All sgRNAs used and genotyping primers are listed on table S2. Dnmt1, Dnmt3a and Dnmt3b triple Knockout cells were obtained from Masaki Okano laboratory.^112^

#### Mitotic errors

Images were captured on a DeltaVision Ultra (Cytiva) microscope system equipped with a 4.2 Mpx sCMOS detector. Fibers were acquired with an ×100 objective (UPlanSApo, 1.4 NA) and 10 × 0.2 μm z section.

#### DNA fiber assay and analysis

To evaluate replication forks via DNA fibers,^113^ exponentially growing cells were pulse-labeled for 20 min with 25 μM 5-iodo-2-deoxyuridine (I7125, Sigma-Aldrich), followed by a second 20-min pulse with 250 μM 5-chloro-2-deoxyuridine (C6891, Sigma-Aldrich). The labeled cells were then washed twice with ice-cold 1X PBS, collected, and suspended at a concentration of 30,000 cells/ml. Subsequently, 30 μL of the suspension was centrifuged onto slides for 4 min at 800 rpm. After cytospin the slides were immersed in Lysis Buffer (0.5% SDS, 200mM Tris-HCl, 50mM EDTA) for 5 min, and DNA molecules were stretched using a homemade LEGO device. DNA fiber spreads were fixed in ice-cold Carnoy fixative for 10 min at room temperature and air-dried. Slides were rehydrated twice in water and incubated for 1 h at room temperature in 2.5 M HCl. Afterward, the slides were rinsed twice in 1X PBS and blocked for 1 h at room temperature in a blocking solution (1X PBS +1% BSA +0.5% Triton X-100 + 0.02% NaN3). The slides were then incubated in primary antibodies overnight at 4°C. The following primary antibodies were used at the indicated dilutions: 1:100 anti-BrdU (BDB347580, Becton Dickson) and 1:250 anti-CldU (ab6326, Abcam). The slides were then rinsed three times in 1X PBS and fixed in 4% paraformaldehyde in PBS for 10 min at room temperature. Afterward, they were rinsed twice in 1X PBS and incubated with 1:1,000 dilutions of Alexa Fluor-conjugated donkey anti-mouse or donkey anti-rat secondary antibodies (Invitrogen) for 2 h at room temperature. Finally, the slides were washed twice in 1X PBS and mounted in ProLong Gold antifade mounting solution. Immunofluorescence images were captured on a DeltaVision Ultra (Cytiva) microscope system equipped with a 4.2 Mpx sCMOS detector. Fibers were acquired with an ×60 objective (PlanApo *N* 1.42 oil) and 1 × 0.2 μm z section. Quantitative image analyses were performed using Fiji (v.2.1.0/1.53c). locking Buffer.

#### RNA seq

RNA extraction was performed using a RNeasy Mini Kit (QIAGEN) using DNase treatment (QIAGEN). RNA was analyzed using a 2100 Bioanalyzer (Aglient Technologies). Libraries with unique adaptor barcodes were multiplexed and sequenced on an NovaSeq 6000 (paired-end, 150 base pair reads). Typical sequencing depth was at least 50 million reads per sample.

#### RNA seq analysis

Quality of datasets was assessed using the FastQC tool. Raw reads were adapter and quality trimmed using Trimgalore.^114^ Reads were aligned to the mouse genome (mm10) with STAR,^115^ using a custom GTF file which contained the NCBI RefSeq genes plus the addition of DFAM’s non-redundant repetitive element annotations.^116^ Optical duplicate reads were filtered using Picard (http://broadinstitute.github.io/picard/). Samtools was used to filter out alignments with MAPQ <30. Count matrices were generated using the featureCounts tool.^117^ DESeq2 was used for the generation of normalized counts, log2FoldChange, and adjusted *p*-values.^118^ baseMean was calculated as the mean of the normalized counts for samples present within a pairwise comparison. MA plots were generated using R and ggplot2.^119^

#### CUT&Tag

CUT&Tag was performed as previously described^59^ with modifications. Briefly, 3×10^^^6 cells were harvested and resuspended in ice-cold nuclei extraction buffer (20mM HEPES KOH pH 7.9, 10mM KCl, 1% Triton X-100, 0.5 mM spermidine, EDTA free protease inhibitor cocktail (Roche)) at left on ice for 10 min. Nuclei were pelleted in a swinging bucket rotor at 1,300xg for 3 min, washed once with PBS and resuspended and cryopreserved in wash buffer 150 (20 mM HEPES pH 7.5, 150 mM NaCl, protease inhibitor cocktail (Roche), 0.5 mM Spermidine) with 10% DMSO, cryovials were placed in −80°C, using in CoolCell freezing containers (Corning) and then stored in liquid nitrogen until experiment. Nuclei were bound to CUTANA Concanavalin A Beads (Epicypher) for 15 min, then incubated with 50 μL Wash125 + 0.1% BSA, 2 mM EDTA, and 1 μL primary antibody targeting either H3K9me3 (Abcam, ab8898) or Flag (Cell Signaling, 2368T) overnight at 4°C. Nuclei were resuspended in 100 μL Wash125 + 1 μL secondary antibody at room temperature for 1 h. Nuclei were washed twice in 1 mL Wash125 (with no Spermidine), then resuspended in 200 μL Wash125 - Spermidine and +0.2% formaldehyde for 2 min and then quenched with 50 μL 2.5 M glycine. Nuclei were washed once in 1 mL Wash350 (20 mM HEPES pH 7.5, 350 mM NaCl, 10 mM NaButyrate, 0.025% Digitonin, protease inhibitor cocktail (Roche), 0.5 mM Sper-midine) then incubated in 47.5 μL Wash350 + 2.5 μL pAG-Tn5 (Epicypher 15–1017) for 1 h. Nuclei were washed twice in 1 mL Wash350, then resuspended in 300 μL Wash350 + 10 mM MgCl2 and incubated for 1 h at 37°C. Tn5 reaction was stopped with 10 μL 0.5 M EDTA, 3 μL 10% SDS, and 3 μL 18 mg/mL Proteinase K, briefly vortexed, then incubated at 55°C for 2 h to reverse crosslinks and release fragments. The fragments were then purified with phenol-chloroform and resuspended in 22 μL 1 mM Tris-HCl pH 8, 0.1 mM EDTA. The entire sample was amplified with Nextera i5 and i7 primers according to the Illumina protocol. The quality of the libraries was assessed using a D1000 ScreenTape on a 2200 TapeStation (Agilent) and quantified using a Qubit dsDNA HS Assay Kit (Thermo Fisher). Libraries with unique adaptor barcodes were multiplexed and sequenced on an NovaSeq 6000 (paired-end, 150 base pair reads). Typical sequencing depth was at least 50 million reads per sample.

#### CUT&Tag analysis

Quality of datasets was assessed using the FastQC tool. Raw reads were adapter and quality trimmed using Trimgalore.^114^ Trimmed reads were aligned to the mouse reference genome (mm10) with Bowtie2^120^ bowtie2 -q -R 3 -N 1 -L 20 -i S,1,0.50 –end-to-end –dove-tail –no-mixed -X 2000). Multimapping reads were randomly assigned. Optical duplicate reads were filtered using Picard. Reads which mapped to the mitochondrial genome were removed with Samtools^121^ (samtools idxstats $sample.sorted.bam | cut -f 1 | grep -v chrM | xargs samtools view -b $sample.sorted.bam). Peak calling was performed with MACS2 software^122^ (–keep-dup all –nomodel -B -f BAMPE, –broad peakcalling was used for H3K9me3, whereas default narrow peaks were called for TASOR-FLAG and) and an FDR cutoff of 0.001 was applied to generate peak bedfiles. Peaks which intersected blacklisted high-signal genomic regions were removed. BigWig files were generated from merged bam files using deepTools^123^ and normalized to counts per million (CPM). Visualization of bigWigs was done in Integrative Genomics Viewer.^108^ Intersections between different peak sets were made using BED-Tools.^108^ Browser-style heatmaps and average profiles were generated using deepTools. Clustered heatmaps (i.e., Figure 3D) were generated using R and pheatmap.^124^

#### ATAC-seq

The modified ATAC-sequencing protocol, Omni-ATAC was performed as previously described.^125^ Briefly, 10^5^ cells were lysed with resuspension buffer (Tris 10 mM, pH 7.4, 10 mM NaCl, 3 mM MgCl2, 0.1% NP-40, 0.1% Tween 20, and 0.01% Digitonin) and nuclei were collected for tagmentation at 37°C for 30 min (Illumina Tagment DNA Enzyme and Buffer Small Kit). The reaction was immediately purified using Qiaquick PCR Purification Kit (Qiagen) and eluted in 20 μL water. Eluted DNA was amplified using NEBNext Ultra II PCR Master Mix (NEB) and purified using AMPure XP beads. Libraries with unique adaptor barcodes were multiplexed and sequenced on an NovaSeq 6000 (paired-end, 150 base pair reads). Typical sequencing depth was at least 50 million reads per sample.

#### ATAC-seq analysis

ATAC-seq data was processed as described above for CUT&Tag.

#### TOBIAS analysis

For TOBIAS analysis, replicate bam files were merged using Samtools. TOBIAS ATACorrect and ScoreBigWig were used to generate scored bigWig files for each merged sample. BINDetect was then used to generate pairwise differential binding scores between samples for each expressed JASPAR motif. For analysis of differential binding scores specifically in promoters, BINDetect was restricted using option –output-peaks to regions of interest, i.e., specific repetitive element subfamilies using bedfiles generated from Dfam’s dfam’s non-redundant hits files (mm10.nrph.hits.gz) or the non-repetitive genome.

### QUANTIFICATION AND STATISTICAL ANALYSIS

All experiments were performed using two or more independent biological replicates, and two or more technical replicates. For viability and fluorescence intensity analyses, after verifying for the assumptions of equal variance and normality, *p* values were calculated using One-Way ANOVA with Tukey’s HSD.^126^ Error bars represent standard deviation. Analyses were performed with Prism (Graphpad).

## Supplementary Material

Supplementary figures

Table S2

Table S1

## Figures and Tables

**Table:** KEY RESOURCES TABLE

REAGENT or RESOURCE	SOURCE	IDENTIFIER
Antibodies		
α-Oct-3/4 (C-10) antibody	Santa Cruz Biotechnology	Cat# sc-5279, RRID:AB_628051
α-Sox-2 (E—4) antibody	Santa Cruz Biotechnology	Cat# sc-365823, RRID:AB_10842165
α-H3K9me3	Cell Signaling Technology	Cat# 13969, RRID:AB_2798355
α-H3K9me3	Abcam	ab8898, RRID:AB_306848
α-Flag	Cell Signaling	2368T, RRID:AB_2217020
α-H3K27me3	Cell Signaling Technology	Cat# 9733, RRID:AB_2616029)
α−5mC	Active Motif	Cat# 39649, RRID:AB_2687950
α−5hmC	Active Motif	Cat# 39092, RRID: AB_2630381
α-Human Gata-6	R&D Systems	Cat# AF1700; RRID:AB_2108901
α-Phospho P-p53 (S15)	Cell Signaling Technology	Cat# 9286, RRID:AB_331741
α-P53 (1C12)	Cell Signaling Technology	Cat# 2524, RRID:AB_331743
α-Phospho-H3 (Serine 10)	Santa Cruz	Cat# sc-374669, RRID:AB_11150094
α-H2AX (γH2AX), (Serine 139)	Abcam	Cat# ab81299, RRID:AB_1640564
α-TASOR (FAM208A)	Atlas Antibodies	Cat# HPA006735, RRID:AB_1852384
α-TASOR (FAM208A)	Atlas Antibodies	Cat# HPA017142, RRID:AB_1852382
α-MPP8	Proteintech	Cat# 16796–1-AP, RRID:AB_2266644
α-Beta Tububilin	Santa Cruz	Cat# sc-5274, RRID:AB_2288090
α-Tububilin	Abcam	Cat# ab6160, RRID:AB_305328
α-P-smad2/3	Cell Signaling Technology	Cat# 8828, RRID:AB_2631089
α-Smad2/3	Abcam	Cat# ab202445
α-Anti-LINE-1 ORF1p	Abcam	Cat# ab216324, RRID:AB_2921327
α-Anti Flag M2	Millipore sigma	Cat# F3165, RRID:AB_259529
α-BrdU	BD Biosciences	Cat# 347580, RRID:AB_10015219
α-CldU	Abcam	Cat# ab6326, RRID:AB_305426
Donkey a-rabbit IgG (H + L)Antibody, Alexa Fluor^™^ 647	Invitrogen	Cat# A31573, RRID: AB_2536183
Donkey a-mouse IgG (H + L) Antibody, Alexa Fluor^™^488	Invitrogen	Cat# A21202, RRID: AB_141607
Donkey a-Goat IgG (H + L) Antibody, Alexa Fluor^™^ 555	Invitrogen	Cat# A-21432, RRID: AB_2535853
Chemicals, peptides, and recombinant proteins		
N2 supplement (100X)	Gibco	Cat. No. 17502–048
B27 supplement (50X)	Gibco	Cat. No. 17504–044
Recombinant Human LIF	Peprotech	Cat. No. 300–05
CHIR-99021	Selleckchem	Cat. No. S1263
PD0325901	Selleckchem	Cat. No. S1036
Recombinant Human FGF-basic	Peprotech	Cat. No. 100–18B
Recombinant Human/Murine/Rat Activin A	Peprotech	Cat. No. 120–14E
Emricasan	Selleckchem	Cat. No. 50–136-5234
endo-IWR 1	Tocris	Cat. No. 3532
XAV-939	Tocris	Cat. No. 3748
BMS493	Tocris	Cat. No. 3509
L-Ascorbic acid 2-phosphate	Sigma-Aldrich	Cat. No. A8960
Dulbecco’s phosphate buffered saline (1X), no calcium, no magnesium (DPBS)	Corning	Cat. No. 354277
DMEM/F12	Gibco	Cat. No. 11320–033
GlutaMAX (100X)	Gibco, Cat. No. 35050–061	Cat. No. 35050–061
MEM Non-Essential Amino Acids (100X)	Gibco	Cat. No. 11140–050
2-Mercaptoethanol (1000X)	Gibco	Cat. No. 21985–023
SYTOX^™^ Green Nucleic Acid Stain	Thermo Fisher	Cat. No. S7020
Geltrex^™^	Thermo Fisher	Cat. No. A1569601
Click-iT^™^ EdU Alexa Fluor^™^ 488 Flow Cytometry Assay Kit	Thermo Fisher	Cat. No. C10420
FxCycle^™^ Far Red	Thermo Fisher	Cat. No. F10348
IM-12	ENZO	Cat# BML-WN102
SB590885	R&D Systems	Cat# 2650/10
WH-4–023	A Chemtek	Cat# 0104–002013
A83–01	Sigma	Cat# SML0788
Chroman1	MedChemExpress	Cat# HY-15392
DNase I	Thermo Fisher Scientific	Cat# 18047019
Deposited data		
ATAC-seq	This Paper	GEO: GSE262193
CUT&TAG	This Paper	GEO: GSE262194
RNA-seq	This Paper	GEO: GSE262195
Experimental models: Cell lines		
Mouse ESCs B6 L4	This Paper	N/A
Mouse ESCs B6 L6	This Paper	N/A
Human ESCs WIBR3	This Paper	RRID:CVCL_9767
Oligonucleotides	Table S2	N/A
Recombinant DNA		
pCAG-PURO-2A-mTASOR	This Paper	N/A
pCAG-PURO-2A-mTASOR-mAID-TAP	This Paper	N/A
pCAG-PURO-2A-mTASOR-TAP	This Paper	N/A
pCAG-PURO-2A-hTASOR isoF-mAID-TAP	This Paper	N/A
pCAG BlastR-T2A-OsTIR1F74G	Adapted from: A. Yesbolatova et al.^37^	N/A

## Highlights

- TASOR is downregulated during the exit of naive pluripotency
- TASOR is essential for cell viability following the exit of naive pluripotency
- TASOR loss leads to unscheduled expression of LINE-1 elements
- LINE-1 upregulation triggers innate immunity in cells exiting naive pluripotency

## References

[1] Silva J, Barrandon O, Nichols J, Kawaguchi J, Theunissen TW, and Smith A (2008). Promotion of Reprogramming to Ground State Pluripotency by Signal Inhibition. PLoS Biol 6, e253. 10.1371/journal.pbio.0060253. 18942890 PMC2570424

[2] Ying QL, Wray J, Nichols J, Batlle-Morera L, Doble B, Woodgett J, Cohen P, and Smith A (2008). The ground state of embryonic stem cell self-renewal. Nature 453, 519–523. 10.1038/nature06968. 18497825 PMC5328678

[3] Takashima Y, Guo G, Loos R, Nichols J, Ficz G, Krueger F, Oxley D, Santos F, Clarke J, Mansfield W, (2015). Resetting Transcription Factor Control Circuitry toward Ground-State Pluripotency in Human. Cell 162, 452–453. 10.1016/j.cell.2015.06.052. 28843285 PMC5628177

[4] Blaschke K, Ebata KT, Karimi MM, Zepeda-Martínez JA, Goyal P, Mahapatra S, Tam A, Laird DJ, Hirst M, Rao A, (2013). Vitamin C induces Tet-dependent DNA demethylation and a blastocyst-like state in ES cells. Nature 500, 222–226. 10.1038/nature12362. 23812591 PMC3893718

[5] Sato N, Meijer L, Skaltsounis L, Greengard P, and Brivanlou AH (2004). Maintenance of pluripotency in human and mouse embryonic stem cells through activation of Wnt signaling by a pharmacological GSK-3-specific inhibitor. Nat. Med 10, 55–63. 10.1038/nm979. 14702635

[6] Rodriguez-Martin B, Alvarez EG, Baez-Ortega A, Zamora J, Supek F, Demeulemeester J, Santamarina M, Ju YS, Temes J, Garcia-Souto D, (2020). Pan-cancer analysis of whole genomes identifies driver rearrangements promoted by LINE-1 retrotransposition. Nat. Genet 52, 306–319. 10.1038/s41588-019-0562-0. 32024998 PMC7058536

[7] Hayashi K, Ohta H, Kurimoto K, Aramaki S, and Saitou M (2011). Reconstitution of the mouse germ cell specification pathway in culture by pluripotent stem cells. Cell 146, 519–532. 10.1016/j.cell.2011.06.052. 21820164

[8] Brons IGM, Smithers LE, Trotter MWB, Rugg-Gunn P, Sun B, Chuva de Sousa Lopes SM, Howlett SK, Clarkson A, Ahrlund-Richter L, Pedersen RA, and Vallier L (2007). Derivation of pluripotent epiblast stem cells from mammalian embryos. Nature 448, 191–195. 10.1038/nature05950. 17597762

[9] Tesar PJ, Chenoweth JG, Brook FA, Davies TJ, Evans EP, Mack DL, Gardner RL, and McKay RDG (2007). New cell lines from mouse epiblast share defining features with human embryonic stem cells. Nature 448, 196–199. 10.1038/nature05972. 17597760

[10] Nichols J, and Smith A (2009). Naive and Primed Pluripotent States. Cell Stem Cell 4, 487–492. 10.1016/j.stem.2009.05.015. 19497275

[11] Nurk S, Koren S, Rhie A, Rautiainen M, Bzikadze AV, Mikheenko A, Vollger MR, Altemose N, Uralsky L, Gershman A, (2022). The complete sequence of a human genome. Science 376, 44–53. 10.1126/science.abj6987. 35357919 PMC9186530

[12] Jachowicz JW, Bing X, Pontabry J, Bošković A, Rando OJ, and Torres-Padilla M-E (2017). LINE-1 activation after fertilization regulates global chromatin accessibility in the early mouse embryo. Nat. Genet 49, 1502–1510. 10.1038/ng.3945. 28846101

[13] Leitch HG, McEwen KR, Turp A, Encheva V, Carroll T, Grabole N, Mansfield W, Nashun B, Knezovich JG, Smith A, (2013). Naive pluripotency is associated with global DNA hypomethylation. Nat. Struct. Mol. Biol 20, 311–316. 10.1038/nsmb.2510. 23416945 PMC3591483

[14] Simon M, Van Meter M, Ablaeva J, Ke Z, Gonzalez RS, Taguchi T, De Cecco M, Leonova KI, Kogan V, Helfand SL, (2019). LINE1 Derepression in Aged Wild-Type and SIRT6-Deficient Mice Drives Inflammation. Cell Metabol 29, 871–885.e5. 10.1016/j.cmet.2019.02.014. PMC6449196 30853213

[15] Della Valle F, Reddy P, Yamamoto M, Liu P, Saera-Vila A, Bensaddek D, Zhang H, Prieto Martinez J, Abassi L, Celii M, (2022). *LINE-1* RNA causes heterochromatin erosion and is a target for amelioration of senescent phenotypes in progeroid syndromes. Sci. Transl. Med 14, eabl6057. 10.1126/scitranslmed.abl6057. 35947677

[16] Rodríguez-Martín C, Cidre F, Fernández-Teijeiro A, Gómez-Mariano G, de la Vega L, Ramos P, Zaballos Á, Monzón S, and Alonso J (2016). Familial retinoblastoma due to intronic LINE-1 insertion causes aberrant and noncanonical mRNA splicing of the RB1 gene. J. Hum. Genet 61, 463–466. 10.1038/jhg.2015.173. 26763876

[17] Tchasovnikarova IA, Timms RT, Matheson NJ, Wals K, Antrobus R, Göttgens B, Dougan G, Dawson MA, and Lehner PJ (2015). GENE SILENCING. Epigenetic silencing by the HUSH complex mediates position-effect variegation in human cells. Science 348, 1481–1485. 10.1126/science.aaa7227. 26022416 PMC4487827

[18] Robbez-Masson L, Tie CHC, Conde L, Tunbak H, Husovsky C, Tchasovnikarova IA, Timms RT, Herrero J, Lehner PJ, and Rowe HM (2018). The HUSH complex cooperates with TRIM28 to repress young retrotransposons and new genes. Genome Res 28, 836–845. 10.1101/gr.228171.117. 29728366 PMC5991525

[19] Tunbak H, Enriquez-Gasca R, Tie CHC, Gould PA, Mlcochova P, Gupta RK, Fernandes L, Holt J, van der Veen AG, Giampazolias E, (2020). The HUSH complex is a gatekeeper of type I interferon through epigenetic regulation of LINE-1s. Nat. Commun 11, 5387. 10.1038/s41467-020-19170-5. 33144593 PMC7609715

[20] Douse CH, Tchasovnikarova IA, Timms RT, Protasio AV, Seczynska M, Prigozhin DM, Albecka A, Wagstaff J, Williamson JC, Freund SMV, (2020). TASOR is a pseudo-PARP that directs HUSH complex assembly and epigenetic transposon control. Nat. Commun 11, 4940. 10.1038/s41467-020-18761-6. 33009411 PMC7532188

[21] Seczynska M, Bloor S, Cuesta SM, and Lehner PJ (2022). Genome surveillance by HUSH-mediated silencing of intronless mobile elements. Nature 601, 440–445. 10.1038/s41586-021-04228-1. 34794168 PMC8770142

[22] Wang X, Xiang Y, Yu Y, Wang R, Zhang Y, Xu Q, Sun H, Zhao ZA, Jiang X, Wang X, (2021). Formative pluripotent stem cells show features of epiblast cells poised for gastrulation. Cell Res 31, 526–541. 10.1038/s41422-021-00477-x. 33608671 PMC8089102

[23] Yang P, Humphrey SJ, Cinghu S, Pathania R, Oldfield AJ, Kumar D, Perera D, Yang JYH, James DE, Mann M, and Jothi R (2019). Multi-omic Profiling Reveals Dynamics of the Phased Progression of Pluripotency. Cell Syst 8, 427–445.e10. 10.1016/j.cels.2019.03.012. 31078527 PMC6544180

[24] Harten SK, Bruxner TJ, Bharti V, Blewitt M, Nguyen TMT, Whitelaw E, and Epp T (2014). The first mouse mutants of D14Abb1e (Fam208a) show that it is critical for early development. Mamm. Genome 25, 293–303. 10.1007/s00335-014-9516-0. 24781204 PMC4105592

[25] Bhargava S, Cox B, Polydorou C, Gresakova V, Korinek V, Strnad H, Sedlacek R, Epp TA, and Chawengsaksophak K (2017). The epigenetic modifier Fam208a is required to maintain epiblast cell fitness. Sci. Rep 7, 9322. 10.1038/s41598-017-09490-w. 28839193 PMC5570896

[26] Müller FJ, Laurent LC, Kostka D, Ulitsky I, Williams R, Lu C, Park IH, Rao MS, Shamir R, Schwartz PH, (2008). Regulatory networks define phenotypic classes of human stem cell lines. Nature 455, 401–405. 10.1038/nature07213. 18724358 PMC2637443

[27] Jinek M, Chylinski K, Fonfara I, Hauer M, Doudna JA, and Charpentier E (2012). A programmable dual-RNA-guided DNA endonuclease in adaptive bacterial immunity. Science 337, 816–821, science. 1225829 [pii]. 10.1126/science.1225829. 22745249 PMC6286148

[28] Yu L, Wei Y, Sun HX, Mahdi AK, Pinzon Arteaga CA, Sakurai M, Schmitz DA, Zheng C, Ballard ED, Li J, (2021). Derivation of Intermediate Pluripotent Stem Cells Amenable to Primordial Germ Cell Specification. Cell Stem Cell 28, 550–567.e12. 10.1016/j.stem.2020.11.003. 33271070

[29] Kinoshita M, Barber M, Mansfield W, Cui Y, Spindlow D, Stirparo GG, Dietmann S, Nichols J, and Smith A (2021). Capture of Mouse and Human Stem Cells with Features of Formative Pluripotency. Cell Stem Cell 28, 453–471.e8. 10.1016/j.stem.2020.11.005. 33271069 PMC7939546

[30] Kojima Y, Kaufman-Francis K, Studdert JB, Steiner KA, Power MD, Loebel DAF, Jones V, Hor A, de Alencastro G, Logan GJ, (2014). The Transcriptional and Functional Properties of Mouse Epiblast Stem Cells Resemble the Anterior Primitive Streak. Cell Stem Cell 14, 107–120. 10.1016/j.stem.2013.09.014. 24139757

[31] Wu J, Okamura D, Li M, Suzuki K, Luo C, Ma L, He Y, Li Z, Benner C, Tamura I, (2015). An alternative pluripotent state confers interspecies chimaeric competency. Nature 521, 316–321. 10.1038/nature14413. 25945737 PMC5278765

[32] Yuan J, and Ofengeim D (2024). A guide to cell death pathways. Nat. Rev. Mol. Cell Biol 25, 379–395. 10.1038/s41580-023-00689-6. 38110635

[33] Lee S, Karki R, Wang Y, Nguyen LN, Kalathur RC, and Kanneganti TD (2021). AIM2 forms a complex with pyrin and ZBP1 to drive PANoptosis and host defence. Nature 597, 415–419. 10.1038/s41586-021-03875-8. 34471287 PMC8603942

[34] Gu Z, Liu Y, Zhang Y, Cao H, Lyu J, Wang X, Wylie A, Newkirk SJ, Jones AE, Lee M, (2021). Silencing of LINE-1 retrotransposons is a selective dependency of myeloid leukemia. Nat. Genet 53, 672–682. 10.1038/s41588-021-00829-8. 33833453 PMC8270111

[35] Li Z, Duan S, Hua X, Xu X, Li Y, Menolfi D, Zhou H, Lu C, Zha S, Goff SP, and Zhang Z (2023). Asymmetric distribution of parental H3K9me3 in S phase silences L1 elements. Nature 623, 643–651. 10.1038/s41586-023-06711-3. 37938774 PMC11034792

[36] Engeland K (2022). Cell cycle regulation: p53-p21-RB signaling. Cell Death Differ 29, 946–960. 10.1038/s41418-022-00988-z. 35361964 PMC9090780

[37] Yesbolatova A, Saito Y, Kitamoto N, Makino-Itou H, Ajima R, Nakano R, Nakaoka H, Fukui K, Gamo K, Tominari Y, (2020). The auxin-inducible degron 2 technology provides sharp degradation control in yeast, mammalian cells, and mice. Nat. Commun 11, 5701. 10.1038/s41467-020-19532-z. 33177522 PMC7659001

[38] Holland AJ, Fachinetti D, Han JS, Cleveland DW Inducible, reversible system for the rapid and complete degradation of proteins in mammalian cells. 109, E3350–E3357 (2012). 10.1073/pnas.1216880109. PMC3523849 23150568

[39] Ayrapetov MK, Gursoy-Yuzugullu O, Xu C, Xu Y, and Price BD (2014). DNA double-strand breaks promote methylation of histone H3 on lysine 9 and transient formation of repressive chromatin. Proc. Natl. Acad. Sci. USA 111, 9169–9174. 10.1073/pnas.1403565111. 24927542 PMC4078803

[40] Zhang L, and Li DQ (2019). MORC2 regulates DNA damage response through a PARP1-dependent pathway. Nucleic Acids Res 47, 8502–8520. 10.1093/nar/gkz545. 31616951 PMC6895267

[41] Gaggioli V, Lo CSY, Reverón-Gómez N, Jasencakova Z, Domenech H, Nguyen H, Sidoli S, Tvardovskiy A, Uruci S, Slotman JA, (2023). Dynamic de novo heterochromatin assembly and disassembly at replication forks ensures fork stability. Nat. Cell Biol 25, 1017–1032. 10.1038/s41556-023-01167-z. 37414849 PMC10344782

[42] Wulfridge P, and Sarma K (2021). A nuclease- and bisulfite-based strategy captures strand-specific R-loops genome-wide. Elife 10, e65146. 10.7554/eLife.65146. 33620319 PMC7901872

[43] Ardeljan D, Steranka JP, Liu C, Li Z, Taylor MS, Payer LM, Gorbounov M, Sarnecki JS, Deshpande V, Hruban RH, (2020). Cell fitness screens reveal a conflict between LINE-1 retrotransposition and DNA replication. Nat. Struct. Mol. Biol 27, 168–178. 10.1038/s41594-020-0372-1. 32042151 PMC7080318

[44] Lehnertz B, Ueda Y, Derijck AAHA, Braunschweig U, Perez-Burgos L, Kubicek S, Chen T, Li E, Jenuwein T, and Peters AHFM (2003). Suv39h-mediated histone H3 lysine 9 methylation directs DNA methylation to major satellite repeats at pericentric heterochromatin. Curr. Biol 13, 1192–1200. 10.1016/s0960-9822(03)00432-9. 12867029

[45] Ren W, Fan H, Grimm SA, Guo Y, Kim JJ, Yin J, Li L, Petell CJ, Tan X-F, Zhang Z-M, (2020). Direct readout of heterochromatic H3K9me3 regulates DNMT1-mediated maintenance DNA methylation. Proc. Natl. Acad. Sci. USA 117, 18439–18447. 10.1073/pnas.2009316117. 32675241 PMC7414182

[46] Rothbart SB, Krajewski K, Nady N, Tempel W, Xue S, Badeaux AI, Barsyte-Lovejoy D, Martinez JY, Bedford MT, Fuchs SM, (2012). Association of UHRF1 with methylated H3K9 directs the maintenance of DNA methylation. Nat. Struct. Mol. Biol 19, 1155–1160. 10.1038/nsmb.2391. 23022729 PMC3492551

[47] Spindel J, Krueger C, Krueger F, Papachristou EK, Kishore K, D’Santos CS, and Reik W (2021). The distinct effects of MEK and GSK3 inhibition upon the methylome and transcriptome of mouse embryonic stem cells. Preprint at bioRxiv, 469000. 10.1101/2021.11.18.469000.

[48] von Meyenn F, Iurlaro M, Habibi E, Liu NQ, Salehzadeh-Yazdi A, Santos F, Petrini E, Milagre I, Yu M, Xie Z, (2016). Impairment of DNA Methylation Maintenance Is the Main Cause of Global Demethylation in Naive Embryonic Stem Cells. Mol. Cell 62, 983. 10.1016/j.molcel.2016.06.005. 27315559 PMC4914604

[49] Gretarsson KH, and Hackett JA (2020). Dppa2 and Dppa4 counteract de novo methylation to establish a permissive epigenome for development. Nat. Struct. Mol. Biol 27, 706–716. 10.1038/s41594-020-0445-1. 32572256

[50] Cimmino L, Neel BG, and Aifantis I (2018). Vitamin C in Stem Cell Reprogramming and Cancer. Trends Cell Biol 28, 698–708. 10.1016/j.tcb.2018.04.001. 29724526 PMC6102081

[51] Brabson JP, Leesang T, Mohammad S, and Cimmino L (2021). Epigenetic Regulation of Genomic Stability by Vitamin C. Front. Genet 12, 675780. 10.3389/fgene.2021.675780. 34017357 PMC8129186

[52] Walter M, Teissandier A, Pérez-Palacios R, and Bourc’his D (2016). An epigenetic switch ensures transposon repression upon dynamic loss of DNA methylation in embryonic stem cells. Elife 5, e11418. 10.7554/eLife.11418. 26814573 PMC4769179

[53] Müller I, Moroni AS, Shlyueva D, Sahadevan S, Schoof EM, Radzisheuskaya A, Højfeldt JW, Tatar T, Koche RP, Huang C, and Helin K (2021). MPP8 is essential for sustaining self-renewal of ground-state pluripotent stem cells. Nat. Commun 12, 3034. 10.1038/s41467-021-23308-4. 34031396 PMC8144423

[54] Mathieu S, Aurélie T, Elena de la M, Mélanie A, Julian I, Fatima El M, Pierre G, Marius W, Sarah K, Berthold G, (2022). DNA methylation restricts coordinated germline and neural fates in embryonic stem cell differentiation. bioRxiv, 513040. 10.1101/2022.10.22.513040.

[55] Pandiloski N, Horvath V, Karlsson OE, Christoforidou G, Dorazehi F, Koutounidou S, Matas J, Gerdes P, Garza R, Jönsson ME, (2023). DNA methylation governs the sensitivity of repeats to restriction by the HUSH-MORC2 corepressor. bioRxiv, 545516. 10.1101/2023.06.21.545516. PMC11364546 39214989

[56] Woodcock DM, Lawler CB, Linsenmeyer ME, Doherty JP, and Warren WD (1997). Asymmetric methylation in the hypermethylated CpG promoter region of the human L1 retrotransposon. J. Biol. Chem 272, 7810–7816. 10.1074/jbc.272.12.7810. 9065445

[57] Seczynska M, and Lehner PJ (2023). The sound of silence: mechanisms and implications of HUSH complex function. Trends Genet 39, 251–267. 10.1016/j.tig.2022.12.005. 36754727

[58] Liu N, Lee CH, Swigut T, Grow E, Gu B, Bassik MC, and Wysocka J (2018). Selective silencing of euchromatic L1s revealed by genome-wide screens for L1 regulators. Nature 553, 228–232. 10.1038/nature25179. 29211708 PMC5774979

[59] Kaya-Okur HS, Wu SJ, Codomo CA, Pledger ES, Bryson TD, Henikoff JG, Ahmad K, and Henikoff S (2019). CUT&Tag for efficient epigenomic profiling of small samples and single cells. Nat. Commun 10, 1930. 10.1038/s41467-019-09982-5. 31036827 PMC6488672

[60] Gerdes P, Chan D, Lundberg M, Sanchez-Luque FJ, Bodea GO, Ewing AD, Faulkner GJ, and Richardson SR (2023). Locus-resolution analysis of L1 regulation and retrotransposition potential in mouse embryonic development. Genome Res 33, 1465–1481. 10.1101/gr.278003.123. 37798118 PMC10620060

[61] Buenrostro JD, Wu B, Chang HY, and Greenleaf WJ (2015). ATAC-seq: A Method for Assaying Chromatin Accessibility Genome-Wide. Curr. Protoc. Mol. Biol 109, 21.29.1–21.29.9. 10.1002/0471142727.mb2129s109. PMC4374986 25559105

[62] Ilık İA, Glažar P, Tse K, Brändl B, Meierhofer D, Müller F-J, Smith ZD, and Aktasx T (2024). Autonomous transposons tune their sequences to ensure somatic suppression. Nature 626, 1116–1124. 10.1038/s41586-024-07081-0. 38355802 PMC10901741

[63] Cosby RL, Judd J, Zhang R, Zhong A, Garry N, Pritham EJ, and Feschotte C (2021). Recurrent evolution of vertebrate transcription factors by transposase capture. Science 371, eabc6405. 10.1126/science.abc6405. 33602827 PMC8186458

[64] Xie HY, Zhang TM, Hu SY, Shao ZM, and Li DQ (2019). Dimerization of MORC2 through its C-terminal coiled-coil domain enhances chromatin dynamics and promotes DNA repair. Cell Commun. Signal. 17, 160. 10.1186/s12964-019-0477-5. 31796101 PMC6892150

[65] Tchasovnikarova IA, Timms RT, Douse CH, Roberts RC, Dougan G, Kingston RE, Modis Y, and Lehner PJ (2017). Hyperactivation of HUSH complex function by Charcot-Marie-Tooth disease mutation in MORC2. Nat. Genet 49, 1035–1044. 10.1038/ng.3878. 28581500 PMC5493197

[66] Matkovic R, Morel M, Lanciano S, Larrous P, Martin B, Bejjani F, Vauthier V, Hansen MMK, Emiliani S, Cristofari G, (2022). TASOR epigenetic repressor cooperates with a CNOT1 RNA degradation pathway to repress HIV. Nat. Commun 13, 66. 10.1038/s41467-021-27650-5. 35013187 PMC8748822

[67] Garland W, Müller I, Wu M, Schmid M, Imamura K, Rib L, Sandelin A, Helin K, and Jensen TH (2022). Chromatin modifier HUSH cooperates with RNA decay factor NEXT to restrict transposable element expression. Mol. Cell 82, 1691–1707.e1698. 10.1016/j.molcel.2022.03.004. 35349793 PMC9433625

[68] Spencley AL, Bar S, Swigut T, Flynn RA, Lee CH, Chen L-F, Bassik MC, and Wysocka J (2023). Co-transcriptional genome surveillance by HUSH is coupled to termination machinery. Mol. Cell 83, 1623–1639.e8. 10.1016/j.molcel.2023.04.014. 37164018 PMC10915761

[69] Zhang S-M, Cai WL, Liu X, Thakral D, Luo J, Chan LH, McGeary MK, Song E, Blenman KRM, Micevic G, (2021). KDM5B promotes immune evasion by recruiting SETDB1 to silence retroelements. Nature 598, 682–687. 10.1038/s41586-021-03994-2. 34671158 PMC8555464

[70] Seth RB, Sun L, Ea CK, and Chen ZJ (2005). Identification and characterization of MAVS, a mitochondrial antiviral signaling protein that activates NF-kappaB and IRF 3. Cell 122, 669–682. 10.1016/j.cell.2005.08.012. 16125763

[71] Mita P, Sun X, Fenyö D, Kahler DJ, Li D, Agmon N, Wudzinska A, Keegan S, Bader JS, Yun C, and Boeke JD (2020). BRCA1 and S phase DNA repair pathways restrict LINE-1 retrotransposition in human cells. Nat. Struct. Mol. Biol 27, 179–191. 10.1038/s41594-020-0374-z. 32042152 PMC7082080

[72] Gasior SL, Wakeman TP, Xu B, and Deininger PL (2006). The human LINE-1 retrotransposon creates DNA double-strand breaks. J. Mol. Biol 357, 1383–1393. 10.1016/j.jmb.2006.01.089. 16490214 PMC4136747

[73] Decombe S, Loll F, Caccianini L, Affannoukoué K, Izeddin I, Mozziconacci J, Escudé C, and Lopes J (2021). Epigenetic rewriting at centromeric DNA repeats leads to increased chromatin accessibility and chromosomal instability. Epigenet. Chromatin 14, 35. 10.1186/s13072-021-00410-x. PMC8317386 34321103

[74] Tatarakis A, Saini H, and Moazed D (2023). Requirements for establishment and epigenetic stability of mammalian heterochromatin. Preprint at bioRxiv. https://10.1101/2023.02.27.530221. 10.1016/j.molcel.2025.08.025 PMC12478525 40972525

[75] Epsztejn-Litman S, Feldman N, Abu-Remaileh M, Shufaro Y, Gerson A, Ueda J, Deplus R, Fuks F, Shinkai Y, Cedar H, and Bergman Y (2008). De novo DNA methylation promoted by G9a prevents reprogramming of embryonically silenced genes. Nat. Struct. Mol. Biol 15, 1176–1183. 10.1038/nsmb.1476. 18953337 PMC2581722

[76] Grewal SIS, and Jia S (2007). Heterochromatin revisited. Nat. Rev. Genet 8, 35–46. 10.1038/nrg2008. 17173056

[77] Saksouk N, Barth TK, Ziegler-Birling C, Olova N, Nowak A, Rey E, Mateos-Langerak J, Urbach S, Reik W, Torres-Padilla ME, (2014). Redundant mechanisms to form silent chromatin at pericentromeric regions rely on BEND3 and DNA methylation. Mol. Cell 56, 580–594. 10.1016/j.molcel.2014.10.001. 25457167

[78] Lukauskas S, Tvardovskiy A, Nguyen NV, Stadler M, Faull P, Ravnsborg T, Özdemir Aygenli B, Dornauer S, Flynn H, Lindeboom RGH, (2024). Decoding chromatin states by proteomic profiling of nucleosome readers. Nature 627, 671–679. 10.1038/s41586-024-07141-5. 38448585 PMC10954555

[79] Chang Y, Sun L, Kokura K, Horton JR, Fukuda M, Espejo A, Izumi V, Koomen JM, Bedford MT, Zhang X, (2011). MPP8 mediates the interactions between DNA methyltransferase Dnmt3a and H3K9 methyltransferase GLP/G9a. Nat. Commun 2, 533. 10.1038/ncomms1549. 22086334 PMC3286832

[80] Zena DJ, Anna EC, Julia RF, and Peter WL (2024). Interplay between Two Paralogous Human Silencing Hub (HuSH) Complexes in Regulating LINE-1 Element Silencing. Preprint at bioRxiv, 573526. 10.1101/2023.12.28.573526. PMC11532391 39489739

[81] Danac JMC, Matthews RE, Gungi A, Qin C, Parsons H, Antrobus R, Timms RT, and Tchasovnikarova IA (2024). Competition between two HUSH complexes orchestrates the immune response to retroelement invasion. Mol. Cell 84, 2870–2881.e5. 10.1016/j.molcel.2024.06.020. 39013473

[82] Ma R, Zhang Y, Zhang J, Zhang P, Liu Z, Fan Y, Wang H-T, Zhang Z, and Zhu B (2024). Targeting pericentric non-consecutive motifs for heterochromatin initiation. Nature 631, 678–685. 10.1038/s41586-024-07640-5. 38961301

[83] Garland W, Müller I, Wu M, Schmid M, Imamura K, Rib L, Sandelin A, Helin K, and Jensen TH (2022). Chromatin modifier HUSH cooperates with RNA decay factor NEXT to restrict transposable element expression. Mol. Cell 82, 1691–1707.e8. 10.1016/j.mol-cel.2022.03.004. 35349793 PMC9433625

[84] Wei J, Yu X, Yang L, Liu X, Gao B, Huang B, Dou X, Liu J, Zou Z, Cui X-L, (2022). FTO mediates LINE1 m6A demethylation and chromatin regulation in mESCs and mouse development. Science 376, 968–973. 10.1126/science.abe9582. 35511947 PMC9746489

[85] Douse CH, Tchasovnikarova IA, Timms RT, Protasio AV, Seczynska M, Prigozhin DM, Albecka A, Wagstaff J, Williamson JC, Freund SM, (2020). TASOR is a pseudo-PARP that directs HUSH complex assembly and epigenetic transposon control. Preprint at bioRxiv, 974832. 10.1101/2020.03.09.974832. PMC7532188 33009411

[86] Holoch D, and Moazed D (2015). RNA-mediated epigenetic regulation of gene expression. Nat. Rev. Genet 16, 71–84. 10.1038/nrg3863. 25554358 PMC4376354

[87] Verdel A, Jia S, Gerber S, Sugiyama T, Gygi S, Grewal SIS, and Moazed D (2004). RNAi-mediated targeting of heterochromatin by the RITS complex. Science 303, 672–676. 10.1126/science.1093686. 14704433 PMC3244756

[88] Schöpp T, Prigozhin DM, Douse C, Kaji K, Cook AG, and O’Carroll D (2023). The DUF3715 domain has a conserved role in RNA-directed transposon silencing. RNA 29, 1471–1480. 10.1261/rna.079693.123. 37433650 PMC10578480

[89] Belgnaoui SM, Gosden RG, Semmes OJ, and Haoudi A (2006). Human LINE-1 retrotransposon induces DNA damage and apoptosis in cancer cells. Cancer Cell Int 6, 13. 10.1186/1475-2867-6-13. 16670018 PMC1464142

[90] Guo Y-L, Gurung C, Fendereski M, and Huang F (2022). Dicer and PKR as Novel Regulators of Embryonic Stem Cell Fate and Antiviral Innate Immunity. J. Immunol 208, 2259–2266. 10.4049/jimmunol.2200042. 35577384 PMC9179006

[91] Hong XX, and Carmichael GG (2013). Innate immunity in pluripotent human cells: attenuated response to interferon-b. J. Biol. Chem 288, 16196–16205. 10.1074/jbc.M112.435461. 23599426 PMC3668775

[92] Kasowitz SD, Ma J, Anderson SJ, Leu NA, Xu Y, Gregory BD, Schultz RM, and Wang PJ (2018). Nuclear m6A reader YTHDC1 regulates alternative polyadenylation and splicing during mouse oocyte development. PLoS Genet 14, e1007412. 10.1371/journal.pgen.1007412. 29799838 PMC5991768

[93] Matsui T, Leung D, Miyashita H, Maksakova IA, Miyachi H, Kimura H, Tachibana M, Lorincz MC, and Shinkai Y (2010). Proviral silencing in embryonic stem cells requires the histone methyltransferase ESET. Nature 464, 927–931. 10.1038/nature08858. 20164836

[94] Karimi MM, Goyal P, Maksakova IA, Bilenky M, Leung D, Tang JX, Shinkai Y, Mager DL, Jones S, Hirst M, and Lorincz MC (2011). DNA Methylation and SETDB1/H3K9me3 Regulate Predominantly Distinct Sets of Genes, Retroelements, and Chimeric Transcripts in mESCs. Cell Stem Cell 8, 676–687. 10.1016/j.stem.2011.04.004. 21624812 PMC3857791

[95] Dodge JE, Kang YK, Beppu H, Lei H, and Li E (2004). Histone H3-K9 methyltransferase ESET is essential for early development. Mol. Cell Biol 24, 2478–2486. 10.1128/mcb.24.6.2478-2486.2004. 14993285 PMC355869

[96] Jiang Q, Ang JYJ, Lee AY, Cao Q, Li KY, Yip KY, and Leung DCY (2020). G9a Plays Distinct Roles in Maintaining DNA Methylation, Retrotransposon Silencing, and Chromatin Looping. Cell Rep 33, 108315. 10.1016/j.celrep.2020.108315. 33113380

[97] Montavon T, Shukeir N, Erikson G, Engist B, Onishi-Seebacher M, Ryan D, Musa Y, Mittler G, Meyer AG, Genoud C, and Jenuwein T (2021). Complete loss of H3K9 methylation dissolves mouse heterochromatin organization. Nat. Commun 12, 4359. 10.1038/s41467-021-24532-8. 34272378 PMC8285382

[98] Peters AH, O’Carroll D, Scherthan H, Mechtler K, Sauer S, Schöfer C, Weipoltshammer K, Pagani M, Lachner M, Kohlmaier A, (2001). Loss of the Suv39h histone methyltransferases impairs mammalian heterochromatin and genome stability. Cell 107, 323–337. 10.1016/s0092-8674(01)00542-6. 11701123

[99] Nicetto D, Donahue G, Jain T, Peng T, Sidoli S, Sheng L, Montavon T, Becker JS, Grindheim JM, Blahnik K, (2019). H3K9me3-heterochromatin loss at protein-coding genes enables developmental lineage specification. Science 363, 294–297. 10.1126/science.aau0583. 30606806 PMC6664818

[100] Bodak M, Cirera-Salinas D, Yu J, Ngondo RP, and Ciaudo C (2017). Dicer, a new regulator of pluripotency exit and LINE-1 elements in mouse embryonic stem cells. FEBS Open Bio 7, 204–220. 10.1002/2211-5463.12174. PMC5292673 28174687

[101] Gao X, Shin YH, Li M, Wang F, Tong Q, and Zhang P (2010). The fat mass and obesity associated gene FTO functions in the brain to regulate postnatal growth in mice. PLoS One 5, e14005. 10.1371/journal.pone.0014005. 21103374 PMC2982835

[102] Yang H, Bai D, Li Y, Yu Z, Wang C, Sheng Y, Liu W, Gao S, and Zhang Y (2022). Allele-specific H3K9me3 and DNA methylation co-marked CpG-rich regions serve as potential imprinting control regions in pre-implantation embryo. Nat. Cell Biol 24, 783–792. 10.1038/s41556-022-00900-4. 35484247

[103] Theunissen TW, Powell BE, Wang H, Mitalipova M, Faddah DA, Reddy J, Fan ZP, Maetzel D, Ganz K, Shi L, (2014). Systematic identification of culture conditions for induction and maintenance of naive human pluripotency. Cell Stem Cell 15, 524–526. 28903030 10.1016/j.stem.2014.09.003 PMC4534765

[104] Yu L, Logsdon D, Pinzon-Arteaga CA, Duan J, Ezashi T, Wei Y, Ribeiro Orsi AE, Oura S, Liu L, Wang L, (2023). Large-scale production of human blastoids amenable to modeling blastocyst development and maternal-fetal cross talk. Cell Stem Cell 30, 1246–1261.e9. 10.1016/j.stem.2023.08.002. 37683605

[105] Chen Y, Tristan CA, Chen L, Jovanovic VM, Malley C, Chu PH, Ryu S, Deng T, Ormanoglu P, Tao D, (2021). A versatile polypharmacology platform promotes cytoprotection and viability of human pluripotent and differentiated cells. Nat. Methods 18, 528–541. 10.1038/s41592-021-01126-2. 33941937 PMC8314867

[106] Yu L, Wei Y, Duan J, Schmitz DA, Sakurai M, Wang L, Wang K, Zhao S, Hon GC, and Wu J (2021). Blastocyst-like structures generated from human pluripotent stem cells. Nature 591, 620–626. 10.1038/s41586-021-03356-y. 33731924

[107] Schindelin J, Arganda-Carreras I, Frise E, Kaynig V, Longair M, Pietzsch T, Preibisch S, Rueden C, Saalfeld S, Schmid B, (2012). Fiji: an open-source platform for biological-image analysis. Nat. Methods 9, 676–682. 10.1038/nmeth.2019. 22743772 PMC3855844

[108] Robinson JT, Thorvaldsdóttir H, Winckler W, Guttman M, Lander ES, Getz G, and Mesirov JP (2011). Integrative genomics viewer. Nat. Biotechnol 29, 24–26. 10.1038/nbt.1754. 21221095 PMC3346182

[109] Pinzon-Arteaga C, Snyder MD, Lazzarotto CR, Moreno NF, Juras R, Raudsepp T, Golding MC, Varner DD, and Long CR (2020). Efficient correction of a deleterious point mutation in primary horse fibroblasts with CRISPR-Cas9. Sci. Rep 10, 7411. 10.1038/s41598-020-62723-3. 32366884 PMC7198616

[110] Wong N, Liu W, and Wang X (2015). WU-CRISPR: characteristics of functional guide RNAs for the CRISPR/Cas9 system. Genome Biol 16, 218. 10.1186/s13059-015-0784-0. 26521937 PMC4629399

[111] Konermann S, Brigham MD, Trevino AE, Joung J, Abudayyeh OO, Barcena C, Hsu PD, Habib N, Gootenberg JS, Nishimasu H, (2015). Genome-scale transcriptional activation by an engineered CRISPR-Cas9 complex. Nature 517, 583–588. 10.1038/nature14136. 25494202 PMC4420636

[112] Tsumura A, Hayakawa T, Kumaki Y, Takebayashi S.i., Sakaue M, Matsuoka C, Shimotohno K, Ishikawa F, Li E, Ueda HR, (2006). Maintenance of self-renewal ability of mouse embryonic stem cells in the absence of DNA methyltransferases Dnmt1, Dnmt3a and Dnmt3b. Gene Cell. 11, 805–814. 10.1111/j.1365-2443.2006.00984.x. 16824199

[113] Parra I, and Windle B (1993). High resolution visual mapping of stretched DNA by fluorescent hybridization. Nat. Genet 5, 17–21. 10.1038/ng0993-17. 8106079

[114] Krueger F Trim Galore: a wrapper tool around Cutadapt and FastQC to consistently apply quality and adapter trimming to FastQ files, with some extra functionality for MspI-digested RRBS-type (Reduced Representation Bisufite-Seq) libraries. http://www.bioinformatics.babraham.ac.uk/projects/trim_galore/.

[115] Dobin A, Davis CA, Schlesinger F, Drenkow J, Zaleski C, Jha S, Batut P, Chaisson M, and Gingeras TR (2013). STAR: ultrafast universal RNA-seq aligner. Bioinformatics 29, 15–21. 10.1093/bioinformatics/bts635. 23104886 PMC3530905

[116] Storer J, Hubley R, Rosen J, Wheeler TJ, and Smit AF (2021). The Dfam community resource of transposable element families, sequence models, and genome annotations. Mobile DNA 12, 2. 10.1186/s13100-020-00230-y. 33436076 PMC7805219

[117] Liao Y, Smyth GK, and Shi W (2014). featureCounts: an efficient general purpose program for assigning sequence reads to genomic features. Bioinformatics 30, 923–930. 10.1093/bioinformatics/btt656. 24227677

[118] Love MI, Huber W, and Anders S (2014). Moderated estimation of fold change and dispersion for RNA-seq data with DESeq2. Genome Biol 15, 550. 10.1186/s13059-014-0550-8. 25516281 PMC4302049

[119] Wickham H (Springer-Verlag New York). ggplot2: Elegant Graphics for Data Analysis}. Springer-Verlag New York.

[120] Langmead B, and Salzberg SL (2012). Fast gapped-read alignment with Bowtie 2. Nat. Methods 9, 357–359. 10.1038/nmeth.1923. 22388286 PMC3322381

[121] Li H, and Durbin R (2009). Fast and accurate short read alignment with Burrows-Wheeler transform. Bioinformatics 25, 1754–1760. 10.1093/bioinformatics/btp324. 19451168 PMC2705234

[122] Zhang Y, Liu T, Meyer CA, Eeckhoute J, Johnson DS, Bernstein BE, Nusbaum C, Myers RM, Brown M, Li W, and Liu XS (2008). Model-based analysis of ChIP-Seq (MACS). Genome Biol 9, R137. 10.1186/gb-2008-9-9-r137. 18798982 PMC2592715

[123] Ramírez F, Ryan DP, Grüning B, Bhardwaj V, Kilpert F, Richter AS, Heyne S, Dündar F, and Manke T (2016). deepTools2: a next generation web server for deep-sequencing data analysis. Nucleic Acids Res 44, W160–W165. 10.1093/nar/gkw257. 27079975 PMC4987876

[124] Kolde R (2019). pheatmap: Pretty Heatmaps.

[125] Corces MR, Trevino AE, Hamilton EG, Greenside PG, Sinnott-Armstrong NA, Vesuna S, Satpathy AT, Rubin AJ, Montine KS, Wu B, (2017). An improved ATAC-seq protocol reduces background and enables interrogation of frozen tissues. Nat. Methods 14, 959–962. 10.1038/nmeth.4396. 28846090 PMC5623106

[126] Kao LS, and Green CE (2008). Analysis of Variance: Is There a Difference in Means and What Does It Mean? J. Surg. Res 144, 158–170. 10.1016/j.jss.2007.02.053. 17936790 PMC2405942

