# Supplementary material for: TASOR expression in naive embryonic stem cells safeguards their developmental potential
Source: Cell Rep. Author manuscript; Available in PMC 2024 Dec 16. (PMC11646706; doi:10.1016/j.celrep.2024.114887)
Supplement: Supplementary figures [file NIHMS2038163-supplement-Supplementary_figures.pdf]

Cell Reports, Volume *43*

Supplemental information

TASOR expression in naive embryonic stem cells safeguards their developmental potential

Carlos A. Pinzon-Arteaga, Ryan O'Hara, Alice Mazzagatti, Emily Ballard, Yingying Hu, Alex Pan, Daniel A. Schmitz, Yulei Wei, Masahiro Sakurai, Peter Ly, Laura A. Banaszynski, and Jun Wu

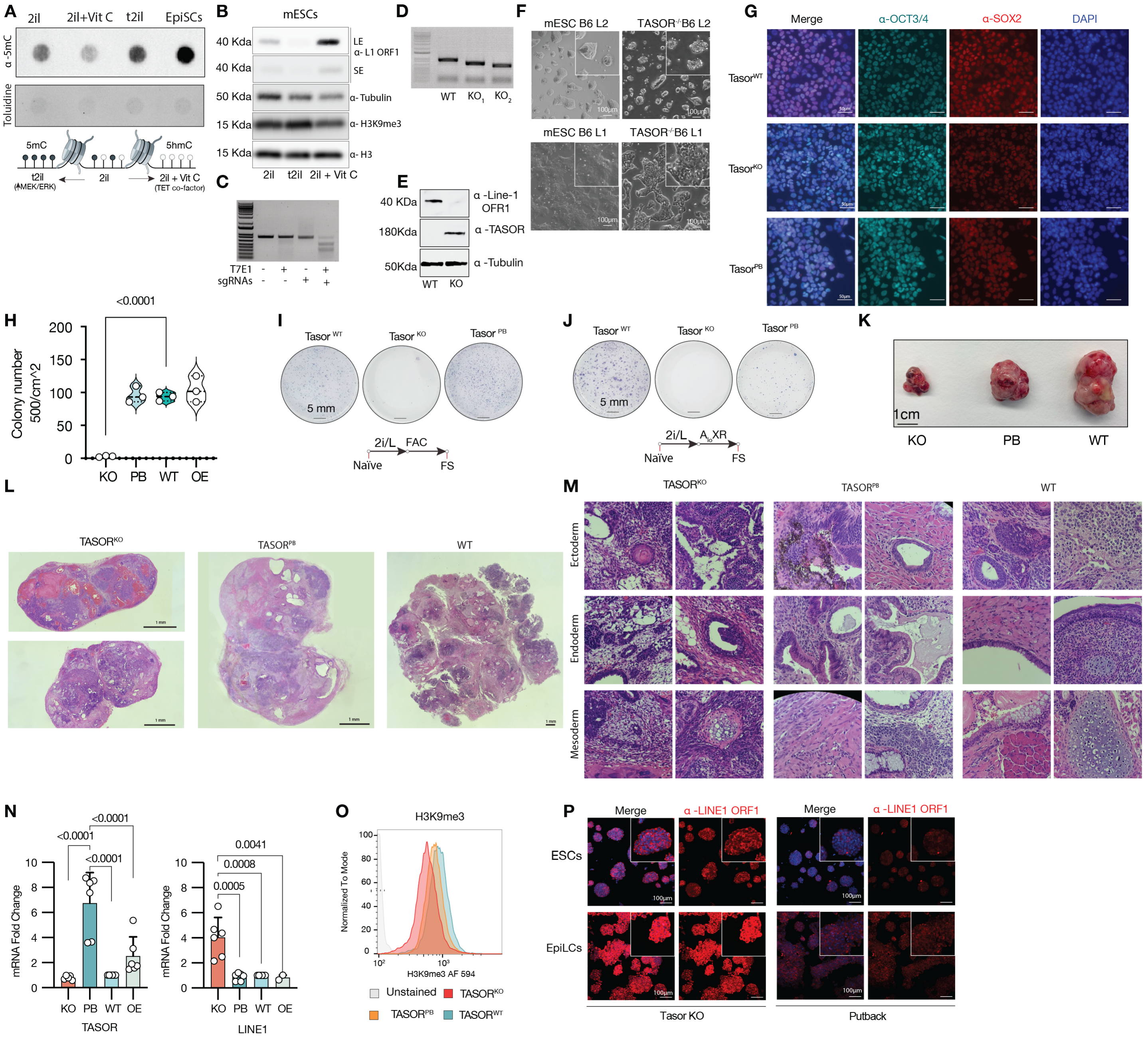

Figure S1. TASOR loss characterization in mouse ESCs. Related to Figure 1.

(A) DNA dot blot for 5mC and toluidine blue staining of genomic DNA from mouse ESCs cultured in 2i/L, PD03 titrated 2i/L (t2i/L), 2i/L plus vitamin C (2i/L +Vit C).

(B) Western blot for L1 ORF1 and H3K9me with and TUBULIN and H3 as loading controls in WT mouse ESCs cultured in 2i/L, t2i/L, and 2i/L +Vit C.

(C) Agarose gel electrophoresis of T7 endonuclease assay for Tasor CRISRPR-CAS9 sgRNAs validation.

(D) Agarose gel electrophoresis of Tasor knockout (KO) genotyping.

(E) Western blot for L1 ORF1, TASOR and α-TUBULIN (loading control) in WT and Tasor KO mouse ESCs.

(F) Brightfield image of TASOR KO lines 2 and line 2. s 1 and 2.

(G) Immunofluorescence staining of OCT4, SOX2 and DAPI, for Tasor WT, KO, and PB cells.

(H) Colony formation quantification of mouse ESCs transition to EpiSCs, cells plated at 500 cells per cm^2 of Tasor WT, KO, and PB cells, dots represent biological replicates. Kruskal-Wallis One-way ANOVA with Tukey's HSD from 3 or more biological replicates.

(I) Brightfield color images of colonies after FAC (also known as FTW) formative cell conversion.

(J) Brightfield color images of colonies after AloXR formative cell conversion.

(K) Representative images of teratomas generated from Tasor KO, PB, and WT mouse ESCs.

(L) A panoramic stitch of brightfield images of hematoxylin and eosin (H&E) stained sections, depicting the overall tissue morphology of Tasor KO, PB, and WT mouse ESC-derived teratomas.

(M H&E staining of Tasor KO, PB, and WT mouse ESC-derived teratomas, showing histological morphology of tissues

representative of ectoderm, endoderm, and mesoderm.
(N) RT-qPCR results for Tasor and L1 in Tasor KO, PB, WT and OE mouse ESCs. Kruskal-Wallis One-way ANOVA with Tukey's HSD from 3 or more biological replicates. Bars represent standard deviation (SD) among biological replicates.
(O) Flow cytometry analysis for global levels of H3K9me3 in Tasor KO, PB, WT and OE mouse ESCs.
(P) Immunofluorescent staining for L1 ORF1 in Tasor Ko and PB mouse ESCs and EpiLCs. Scale bar – 100 μm.

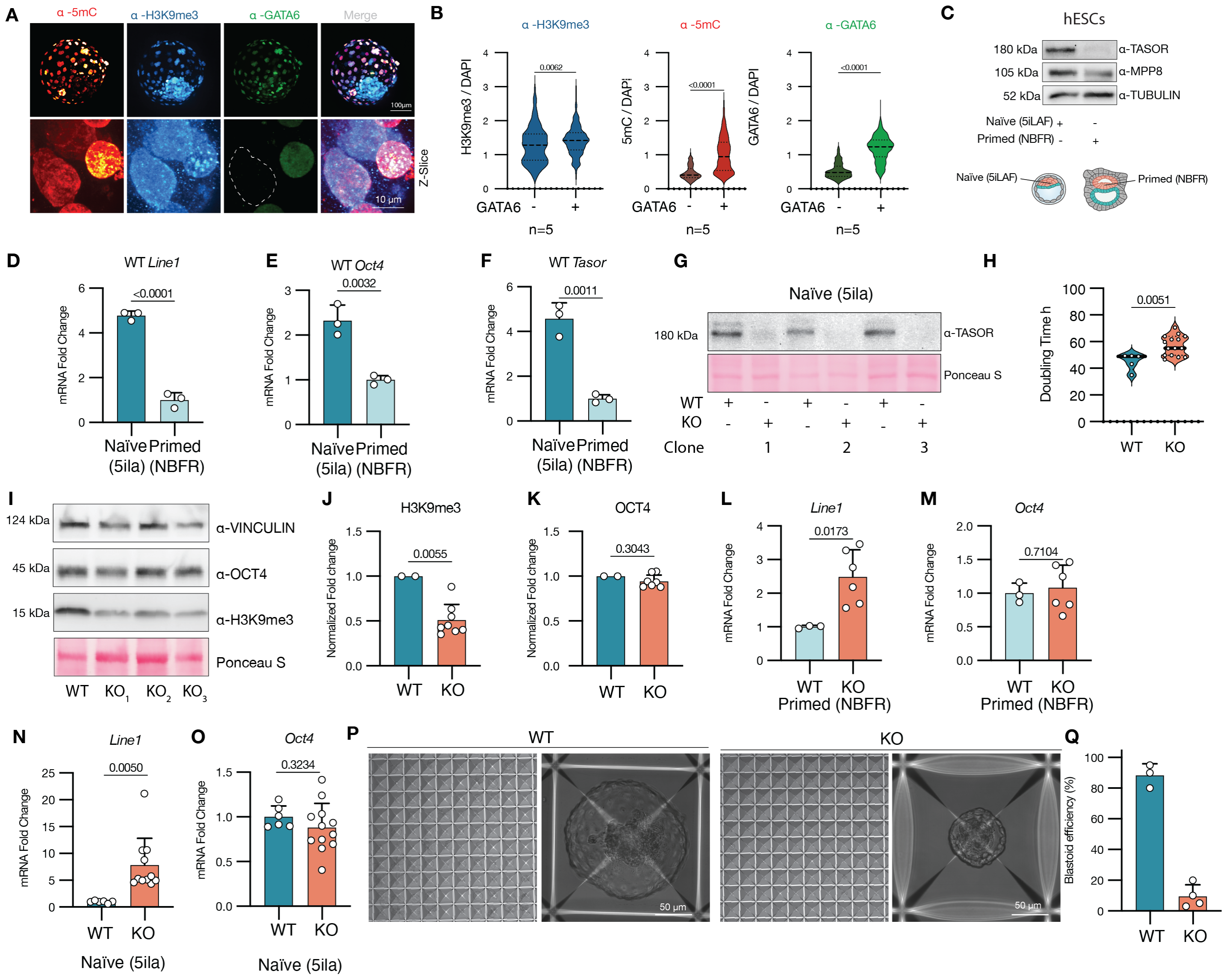

Figure S2 TASOR loss characterization in human ESCs. Related to Figure 1.

(A) Representative immunofluorescent staining of human blastoids derived from naïve human ESCs for GATA6, H3K9me3, and DNA 5mC methylation.

(B) Quantification of H3K9me3, 5mC and GATA6, comparing GATA6 positive (Trophectoderm and hypoblast) and GATA6 negative (Epiblast) cells in human blastoids, depicting 5mC methylation gain upon exit of naïve pluripotency. Unpaired t Test from 5 biological replicates.

(C) Western blot showing the expression of TASOR, MPP8 and α-TUBULIN (loading control) in human ESCs cultured in 5i/la (Naïve) and NBFR (Primed) conditions.

(D-F) RT-qPCR mRNA fold change between naïve (5iLA) and primed (NBFR) human WIBR3 ΔPE-OCT4 GFP ESCs for (D) L1 (L1P), (E) OCT4, and (F) TASOR. Unpaired t Test from 3 or more biological replicates. Bars represent standard deviation (SD) among biological replicates.

(G) Western blot for TASOR knockout clones in naïve human ESCs.

(H) Doubling time between Tasor Wild type (WT) and knockout (KO) cells.

(I) Western blot analysis for OCT4 and H3K9me3 in three different TASOR KO clones and WT human naïve WIBR3 ΔPE-OCT4 GFP ESCs.

(J) Loading control normalized relative protein levels of H3K9me3 between TASOR KO and WT naïve human ESCs. Unpaired t Test from 3 or more biological replicates. Bars represent standard deviation (SD) among biological replicates.

(K) Loading control normalized relative protein levels of OCT4 between TASOR KO and WT naïve human ESCs. Unpaired t Test from 3 or more biological replicates. Bars represent standard deviation (SD) among biological replicates.

(L) RT-qPCR analysis showing mRNA fold change for L1(L1P) between TASOR KO and WT primed human ESCs. Unpaired t Test from 3 or more biological replicates. Bars represent standard deviation (SD) among biological replicates.

(M) RT-qPCR analysis showing mRNA fold change for OCT4 between TASOR KO and WT primed human ESCs. Unpaired t Test from 3 or more biological replicates. Bars represent standard deviation (SD) among biological replicates.

(N) RT-qPCR analysis showing mRNA fold change for L1 between TASOR KO and WT naïve human ESCs. Unpaired t Test

from 3 or more biological replicates. Bars represent standard deviation (SD) among biological replicates.

(O) RT-qPCR analysis showing mRNA fold change for OCT4 between TASOR KO and WT naïve human ESCs. Unpaired t Test from 3 or more biological replicates. Bars represent standard deviation (SD) among biological replicates.

(P) Brightfield image of Aggrewell plate showing blastoid formation efficiencies from TASOR KO and WT naïve human ESCs.

(Q) Quantification of blastoid formation efficiency from TASOR KO and WT naïve human ESCs.

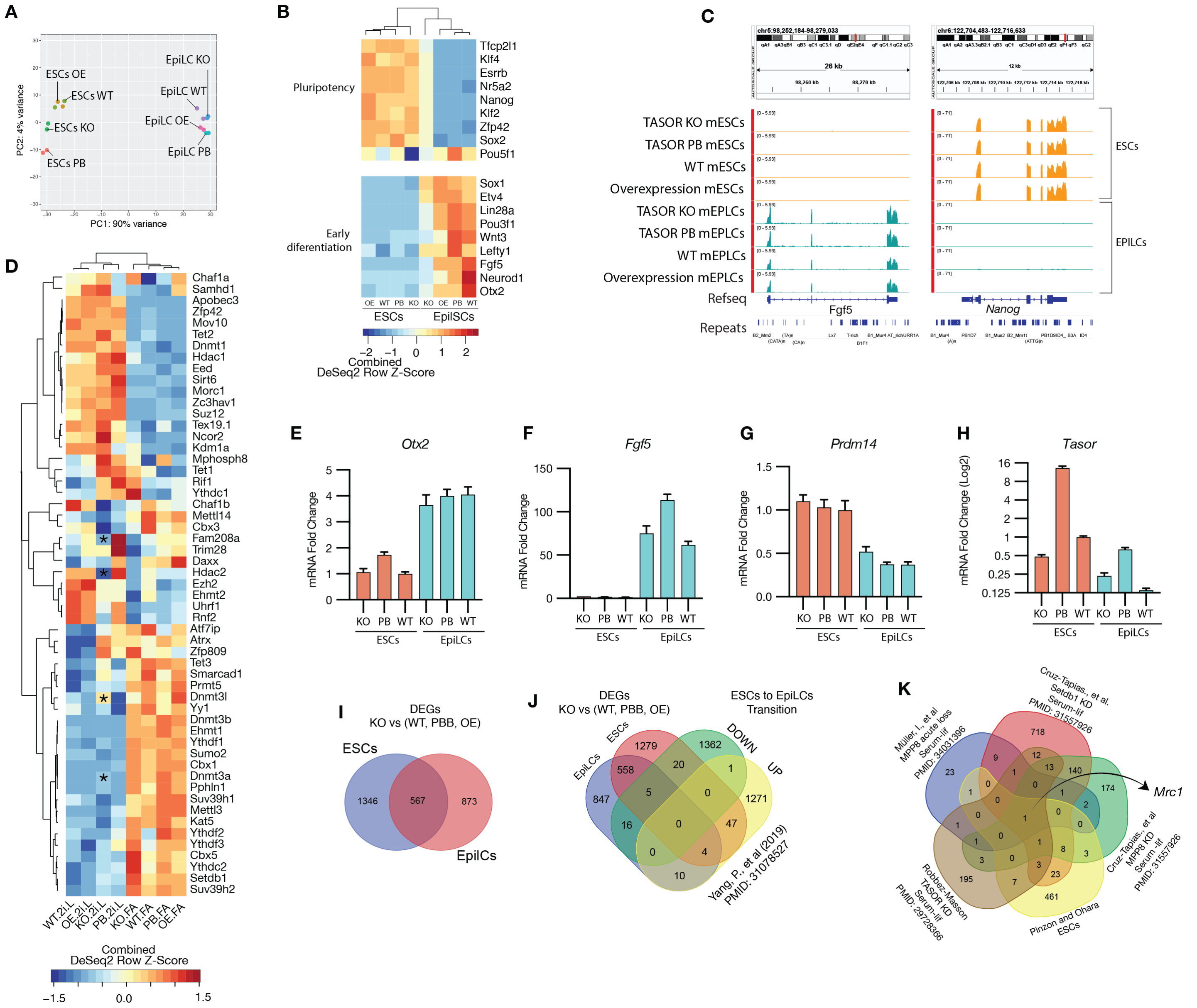

Figure S3. RNA-seq characterization of mouse ESCs and EpiLCs upon TASOR loss. Related to Figure 1.

(A) Principal component analysis of mouse ESCs and EpiLCs samples.

(B) Heatmap showing various pluripotency and early differentiation markers in Tasor KO, PB, WT, OE mouse ESC and EpiLCs.

(C) IGV RNA-seq tracks for Tasor knockout (KO), putback (PB), and Wild type (WT), overexpression (OE) mouse ESC (2i/L) and EpiLCs (FA) depicting expression of Fgf5 and Nanog.

(D) Heatmap showing various epigenetic markers in Tasor KO, PB, WT, OE mouse ESC and EpiLCs.

(E) RT-qPCR analysis showing mRNA fold change for Otx2 in WT, Tasor KO and PB mouse ESCs and EpiLCs. Bars represent standard deviation (SD) among 2 or more biological replicates.

(F) RT-qPCR analysis showing mRNA fold change for Fgf5 in WT, Tasor KO and PB mouse ESCs and EpiLCs. Bars represent standard deviation (SD) among 2 or more biological replicates.

(G) RT-qPCR analysis showing mRNA fold change for Prdm14 in WT, Tasor KO and PB mouse ESCs and EpiLCs. Bars represent standard deviation (SD) among 2 or more biological replicates.

(H) RT-qPCR analysis showing mRNA fold change for Tasor in WT, KO and PB mouse ESCs and EpiLCs.

(I) Venn diagram showing differentially expressed genes (DEGs) between Tasor KO versus WT, PB, and OE in mouse ESCs and EpiLCs.

(J) Venn diagram showing DEGs in mouse ESCs and EpiLCs, compared to upregulated and downregulated genes during the transition from mouse ESCs to EpiLCs using the stem cell atlas database[23].

(K) Venn diagram showing the common upregulated genes between our datasets and those from Cruz-Tapias., et al (Mpp8 knockdown and Setdb1 knockdown)[132], Müller, I., et al (Mpp8 acute loss)[52] and Robbez-Masson[18] (Tasor Knockdown).

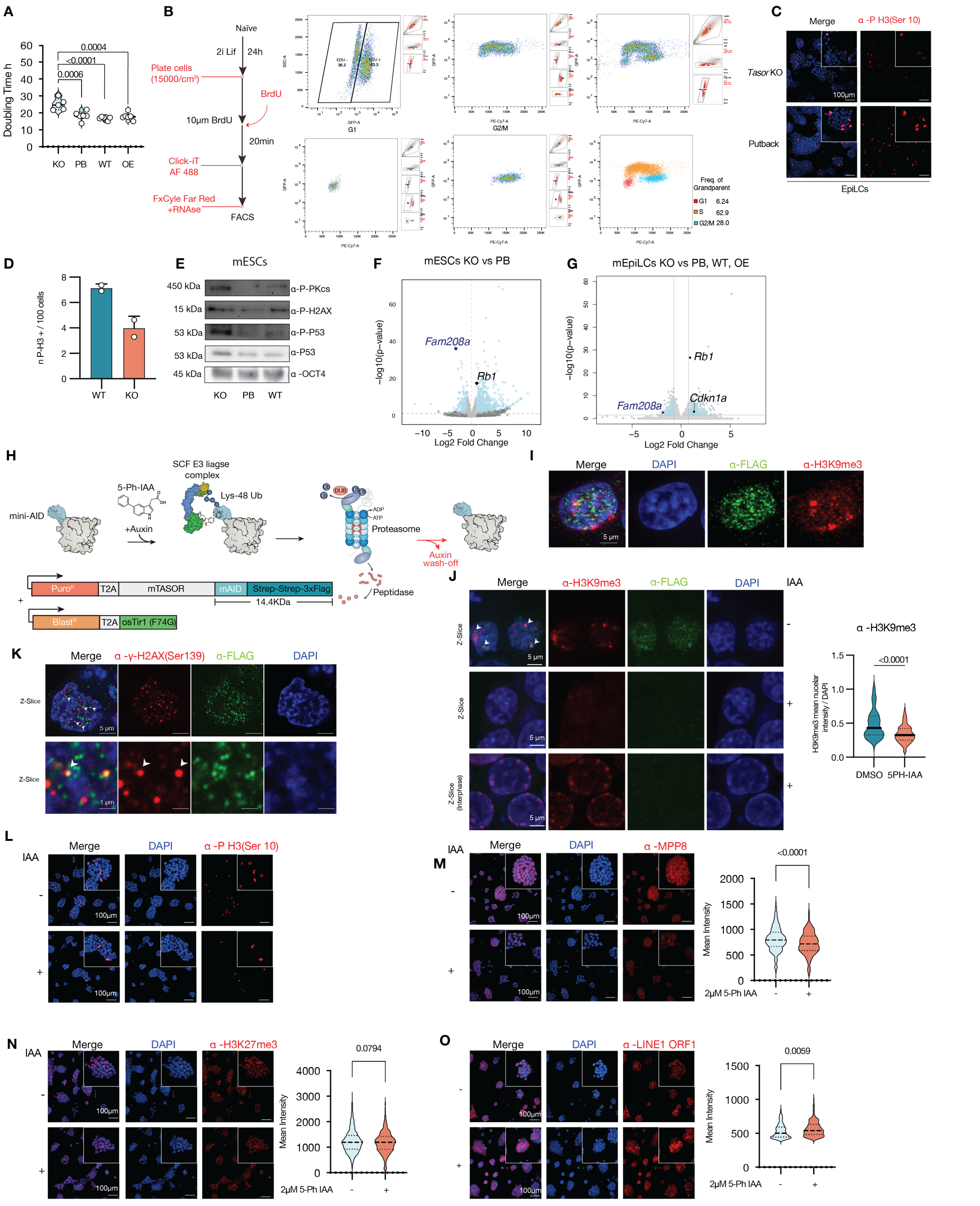

Figure S4. TASOR loss induces DNA damage and cell cycle arrest. Related to Figure 2.

(A) Doubling time of Tasor KO, PB, WT and OE mouse ESCs.

(B) Diagram for cell cycle analysis using BrdU incorporation and Click-it AF488 staining with FxCycle far-red DNA stain. The gating strategy to separate S, G1, and G2/M populations is shown, with percentages of the grandparent population

indicated.
(C) Immunofluorescence staining for the mitosis marker phospho H3 (Serine 10) of Tasor KO EpiLCs.
(D) Quantification of phospho H3 positive mESCs per 100 cells. Bars represent standard deviation (SD) among 2 or more biological replicates.
(E) Western blot for DNA damage markers: phospho DNA protein kinase catalytic subunit (DNAPKs) serine 2056, phosphoserine 139 of histone H2AX (γH2AX), and phosphoserine 15 of P-53 in Tasor KO, PB and WT mouse ESCs.
(F) Volcano plot comparing Tasor KO and PB mouse ESCs, highlighting the upregulation of the Rb1 transcript.
(G) Volcano plot comparing Tasor KO with PB, WT and OE EpiLCs, highlighting the de-repression of Rb1 and Cdkn1a (p21).
(H) Diagram of the auxin-inducible degron 2 constructs.
(I) Z-slice confocal immunofluorescence image showing H3K9me3 and FLAG staining; white arrows indicate colocalization spots.
(J) Z-slice confocal image of immunofluorescence staining for H3K9me3 and FLAG with DAPI counterstaining for TASOR-mAID-TAP. The bottom panel depicts interphase cells, showing H3K9me3 chromocenters associated with the nuclear lamina. Unpaired t Test from 2 or more biological replicates.
(K) Z-slice confocal image of immunofluorescence staining for phosphoserine 139 of histone H2AX (γH2AX) and FLAG, with DAPI counterstaining for TASOR-mAID-TAP.
(L-O) Maximum intensity projection immunofluorescence staining in Tasor KO mouse ESCs rescued with TASOR-mAID-TAP, with or without 2μM 5-Ph IAA treatment for 48 hours. Staining was performed for (L) phospho H3, (M) MPP8, (N) H3K27me3, and (O) L1 ORF1. Unpaired t Test from 2 or more biological replicates.

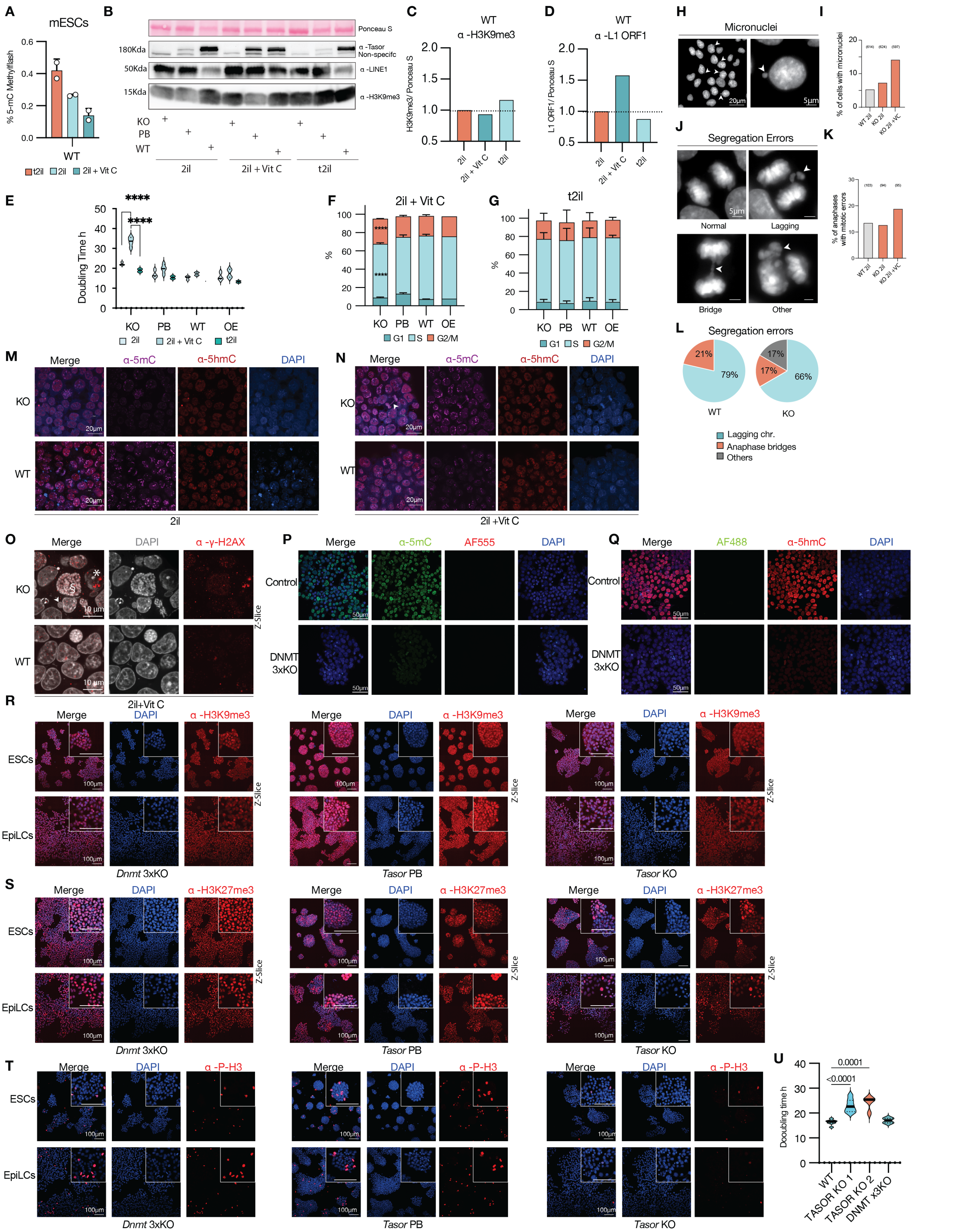

Figure S5. DNA methylation loss does not phenocopy TASOR loss, but Tasor KO mouse ESCs are sensitized to vitamin C-induced hypomethylation. Related to Figure 2.

(A) Methylflash quantification of 5mC levels in mouse ESCs cultured in 2i/L (1μM PD03), t2i/L (0.3μM PD03) and 2i/L with vitamin C (100μg/ml). Bars represent standard deviation (SD) among 2 or more biological replicates.
(B) Western blot for H3K9me3, L1 ORF-1 and TASOR in Tasor KO, PB, and WT mouse ESCs cultured in 2i/L, t2i/L and 2i/L with vitamin C, Ponceu S is shown as loading control.
(C) Quantification of H3K9me3 signal normalized to Ponceau S in WT mouse ESCs.
(D) Quantification of L1 ORF-1 signal normalized to Ponceau S in WT mouse ESCs.
(E)Doubling time quantification in Tasor KO, PB, WT, and OE mouse ESCs cultured in 2i/L, t2i/L and 2i/L with vitamin C. Kruskal-Wallis One-way ANOVA with Tukey's HSD from 2 or more biological replicates, adjusted value (p) $p<0.05$ shown as *, $p<0.001$ shown as **, $p<0.0001$ shown as ***.
(F) Flow cytometry cell cycle analysis via EdU incorporation and DNA staining of Tasor KO, PB, WT, and overexpression (OE) mouse ESCs in 2il + vitamin C (100μg/ml). Kruskal-Wallis One-way ANOVA with Tukey's HSD from 2 or more biological replicates, adjusted value (p) $p<0.05$ shown as *, $p<0.001$ shown as **, $p<0.0001$ shown as ***. Bars represent standard deviation (SD) among biological replicates.
(G) Flow cytometry cell cycle analysis via EdU incorporation and DNA staining of Tasor KO, PB, WT, and overexpression (OE) mouse ESCs in t2i/L (0.3μM PD03). Kruskal-Wallis One-way ANOVA with Tukey's HSD from 2 or more biological replicates, adjusted value (p) $p<0.05$ shown as *, $p<0.001$ shown as **, $p<0.0001$ shown as ***. Bars represent standard deviation (SD) among biological replicates.
(H) Confocal epifluorescence images depicting different types of micronuclei.
(I) Quantification of the percentage of cells with micronuclei between Tasor KO and WT mouse ESCs in 2i/L or 2i/L plus vitamin C.
(J)Confocal epifluorescence images depicting different types of segregation errors.
(K) Quantification of the percentage of anaphases with mitotic errors between Tasor KO and WT mouse ESCs in 2i/L or 2i/L plus vitamin C.
(L) Quantification of segregation errors between Tasor KO and WT mouse ESCs.
(M) Immunofluorescence staining for 5mC and 5hmC with DAPI counterstaining of Tasor KO and WT mouse ESCs cultured in 2i/L.
(N) Immunofluorescence staining for 5mC and 5hmC with DAPI counterstaining of Tasor KO and WT mouse ESCs cultured in 2i/L plus vitamin C.
(O) Z-slice confocal immunofluorescence for phosphoserine 139 of histone H2AX (γH2AX) with DAPI counterstaining of Tasor KO mouse ESCs cultured in 2i/L plus vitamin C, depicting DNA damage (*), micronuclei (arrowhead), and accumulation of abnormal karyotypes (§).
(P) Immunofluorescence staining for 5mC with DAPI counterstaining in Dnmt1, Dnmt3a and Dnmt3b triple knockout (Dnmt x3KO) and WT mouse ESCs.
(Q) Immunofluorescence staining for 5hmC with DAPI counterstaining in Dnmt x3KO and WT mouse ESCs.
(R) Z-slice confocal immunofluorescence staining for H3K9me3 in Dnmt x3KO, Tasor KO, and Tasor PB mouse ESCs and EpiLCs.
(S) Z-slice confocal immunofluorescence staining for H3K27me3 in Dnmt x3KO, Tasor KO, and Tasor PB mouse ESCs and EpiLCs.
(T) Immunofluorescence staining for the mitosis marker phospho H3 (Serine 10) in Dnmt x3KO, Tasor KO, and Tasor PB mouse ESCs and EpiLCs.
(U) Doubling time of WT, Tasor KO and Dnmt x3KO mouse ESCs. Kruskal-Wallis One-way ANOVA with Tukey's HSD from 2 or more biological replicates.

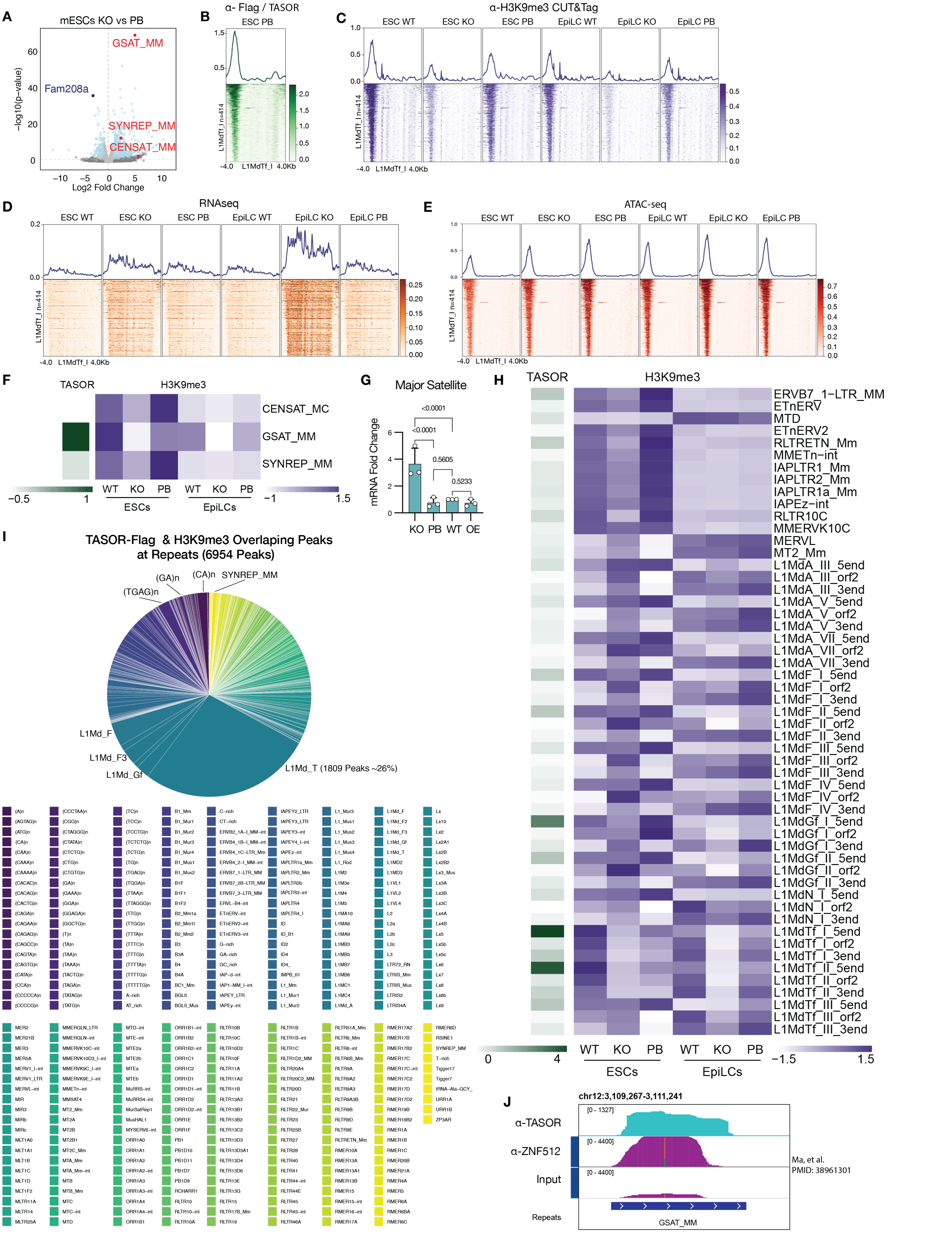

Figure S6. Epigenomic profiling of H3K9me3 and TASOR-3xFLag reveals TASOR regulation of L1 and repeats. Related to Figure 3.

(A) Volcano plot comparing Tasor KO and PB mouse ESCs, showing the derepression of General satellite repeats (GSAT_MM), Simple or minor Repeats (SYNREP_MM), and Centromeric satellite repeats (CENSAT_MC).
(B) Average CUT&Tag profiles (top) and heatmaps (bottom) at L1MdTf_I (n=414) for TASOR-3xFlag.
(C) Average CUT&Tag profiles (top) and heatmaps (bottom) at L1MdTf_I (n=414) for H3K9me3.
(D) Average RNAseq profiles (top) and heatmaps (bottom) at L1MdTf_I (n=414).
(E) Average ATACseq profiles (top) and heatmaps (bottom) at L1MdTf_I (n=414) for H3K9me3.
(F) Heatmap for CUT&Tag analysis of TASOR-3xFlag and H3K9me3 at General satellite repeats (GSAT_MM), Simple or minor Repeats (SYNREP_MM), and Centromeric satellite repeats (CENSAT_MC).
(G) RT-qPCR analysis showing fold change for Major satellite repeats RNA between Tasor KO, PB, WT mouse ESCs. Kruskal-Wallis One-way ANOVA with Tukey's HSD from 2 or more biological replicates.
(H) Heatmap for CUT&Tag analysis of TASOR-3xFlag and H3K9me3 across different retroviral families.
(I) Pie chart showing the overlap of H3K9me3 and TASOR-Flag CUT&Tag peaks mapped to repetitive elements.
(J) IGV tracks at General satellite repeats (GSAT_MM) for Cut&Tag for TASOR-3xFLAG(teal), and CHIPseq for ZNF512 (Magenta) from Ma, et al[81] in mouse ESCs.

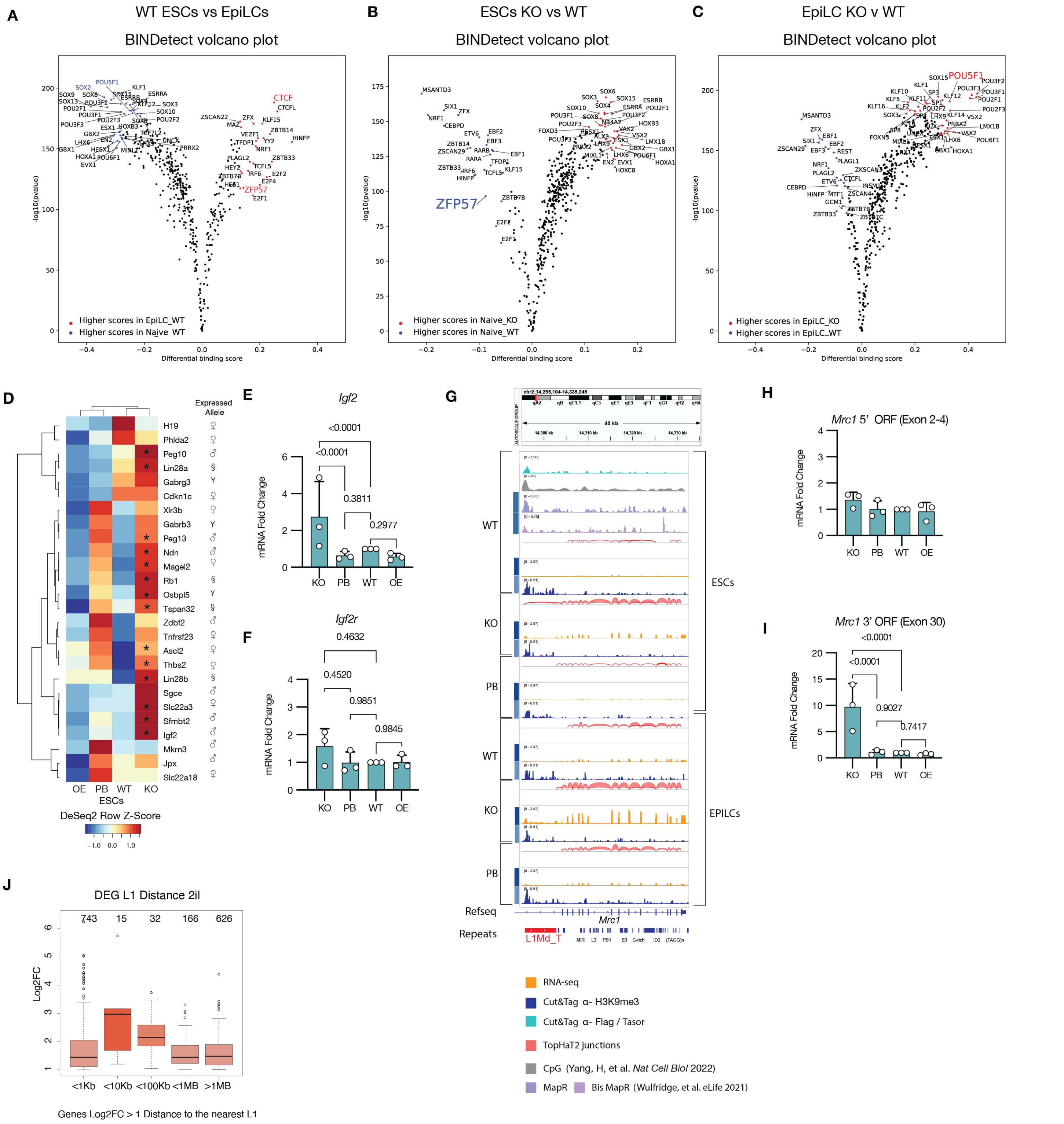

Figure S7. Analysis of differentially expressed genes and ATAC peaks in Tasor KO mouse ESCs. Related to Figure 3.
(A) Differential transcription factor binding scores from ATAC-seq data using BINDetect, comparing mouse ESCs and EpiLCs.
(B) Differential transcription factor binding scores from ATAC-seq data using BINDetect, comparing Tasor KO and WT mouse ESCs.
(C) Differential transcription factor binding scores from ATAC-seq data using BINDetect, comparing Tasor KO and WT EpiLCs.
(D) Heatmap of different differentially expressed imprinted genes in mouse ESC. * Represent significant differentially expressed genes, log2 fold change ≥1, pval≤0.05, § Predicted or isoform dependent, ¥ Conflicting data.
(E) RT-qPCR analysis showing mRNA fold change for Igf2 between Tasor KO, PB, WT mouse ESC. Kruskal-Wallis One-way ANOVA with Tukey's HSD from 2 or more biological replicates. Bars represent standard deviation (SD) among biological

replicates.

(F) RT-qPCR analysis showing mRNA fold change for IGF2 receptor Igf2r between Tasor KO, PB, WT mouse ESCs. Kruskal-Wallis One-way ANOVA with Tukey's HSD from 2 or more biological replicates. Bars represent standard deviation (SD) among biological replicates.

(G) IGV tracks for RNA-seq (orange), Cut&Tag tracks for TASOR-3xFLAG(teal), H3K9me3 (dark blue), ToPHAT2 junctions(red), CpG number from Yang, et al [101] and R-loops from Wulfridge, et al[41], in Tasor KO, PB, WT mouse ESC and EpiLCs, depicting the position effect varigenation of L1s affecting nearby gene expression upon TASOR loss.

(H) RT-qPCR analysis showing mRNA fold change for Mrc1 5' ORF (Exon 2-3) between Tasor KO, PB, WT mouse ESCs. Kruskal-Wallis One-way ANOVA with Tukey's HSD from 2 or more biological replicates. Bars represent standard deviation (SD) among biological replicates.

(I) RT-qPCR analysis showing mRNA fold change for Mrc1 3' ORF (Exon 30) between Tasor KO, PB, WT mouse ESCs. Kruskal-Wallis One-way ANOVA with Tukey's HSD from 2 or more biological replicates. Bars represent standard deviation (SD) among biological replicates.

(J) The proximity of upregulated DEG genes to L1 elements in mouse ESCs. Bars represent standard deviation (SD).

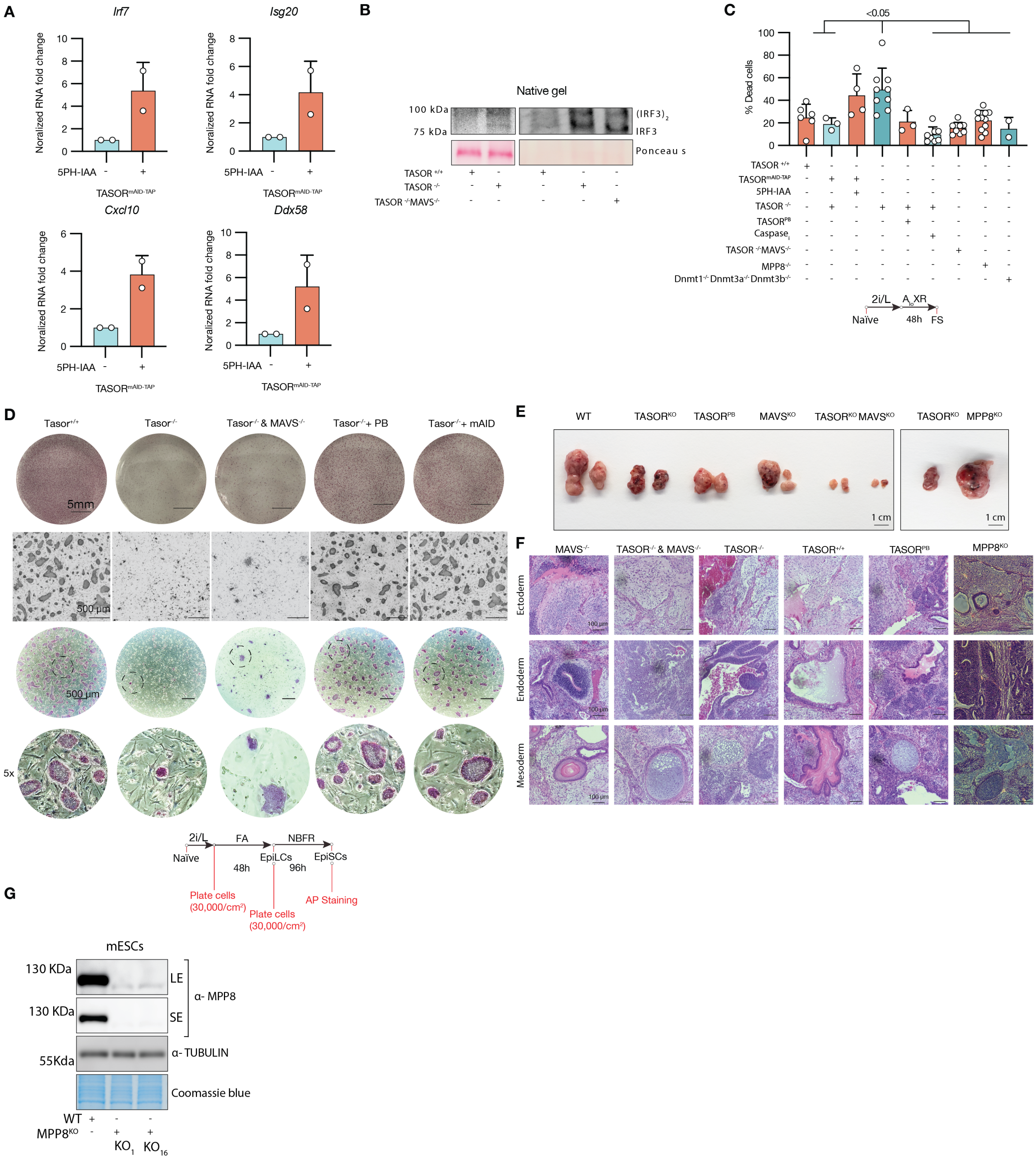

Figure S8. An innate immune response mediates cell death upon Tasor KO ESC-to-EpiLC transition. Related to Figure 4.

(A) RT-qPCR analysis of innate immune response genes in TASOR mAID mouse ESCs with and without 2μM 5ph-IAA treatment. Bars represent standard deviation (SD) among 2 biological replicates.

(B) Western blot analysis using native PAGE gel for checking IRF3 dimerization in WT, Tasor KO, Mpp8 KO and Tasor/Mpp8 dKO mouse ESCs.

(C) Flow cytometry quantification of the percentage of dead cells using SYTOX green staining. Bars represent standard deviation (SD), Kruskal-Wallis One-way ANOVA with Tukey's HSD from 2 or more biological replicates.

(D) Alkaline phosphatase staining of colony formation assay for Tasor WT, KO, and TB, Tasor KO rescued with TASOR-mAID-TAP , and Tasor/Mavs dKO mouse ESCs during transition to EpiSCs.

(E) Teratomas generated from Tasor WT, KO, PB, Mavs KO, Tasor/Mavs dKO, and Mpp8 KO mouse ESCs.

(F) H&E staining of teratomas derived from Tasor WT, KO, PB, Mavs KO, Tasor/Mavs dKO, and Mpp8 KO mouse ESCs, showing histological morphology of tissues representative of ectoderm, endoderm, and mesoderm. Scale bar: 100μm.
(G) Western blot analysis for MPP8 in WT and two different Mpp8 KO clones of mouse ESCs. LE: Long Exposure, SE: Short Exposure.

SUPPLEMENTAL REFERENCENCES

127. Veazey K.J., Golding M.C. Selection of stable reference genes for quantitative rt-PCR comparisons of mouse embryonic and extra-embryonic stem cells. PLoS One 2011;6:e27592. https://doi.org/10.1371/journal.pone.0027592

128. Dunican D.S., Cruickshanks H.A., Suzuki M., Semple C.A., Davey T., Arceci R.J., Greally J., Adams I.R., Meehan R.R. Lsh regulates LTR retrotransposon repression independently of Dnmt3b function. Genome Biol. 2013;14:R146. https://doi.org/10.1186/gb-2013-14-12-r146

129. Abraham J., Prajapati S.I., Nishijo K., Schaffer B.S., Taniguchi E., Kilcoyne A., McCleish A.T., Nelon L.D., Giles F.G., Efstratiadis A., et al. Evasion Mechanisms to Igf1r Inhibition in Rhabdomyosarcoma. Mol. Cancer Therapeut. 2011;10:697–707. https://doi.org/10.1158/1535-7163.mct-10-0695

130. Linneberg-Agerholm M., Wong Y.F., Romero Herrera J.A., Monteiro R.S., Anderson K.G.V., Brickman J.M. Naïve human pluripotent stem cells respond to Wnt, Nodal and LIF signalling to produce expandable naïve extra-embryonic endoderm. Development 2019;146:dev180620. https://doi.org/10.1242/dev.180620

131. Montoya-Durango D.E., Liu Y., Teneng I., Kalbfleisch T., Lacy M.E., Steffen M.C., Ramos K.S. Epigenetic control of mammalian LINE-1 retrotransposon by retinoblastoma proteins. Mutat. Res. 2009;665:20–28. https://doi.org/10.1016/j.mrfmmm.2009.02.011

132. Cruz-Tapias P., Robin P., Pontis J., Maestro L.D., Ait-Si-Ali S. The H3K9 Methylation Writer SETDB1 and its Reader MPP8 Cooperate to Silence Satellite DNA Repeats in Mouse Embryonic Stem Cells. Genes 2019;10:750. https://doi.org/10.3390/genes10100750
